# Years of good life is a well-being indicator designed to serve research on sustainability

**DOI:** 10.1073/pnas.1907351118

**Published:** 2021-03-15

**Authors:** Wolfgang Lutz, Erich Striessnig, Anna Dimitrova, Simone Ghislandi, Anastasia Lijadi, Claudia Reiter, Sonja Spitzer, Dilek Yildiz

**Affiliations:** ^a^Department of Demography, University of Vienna, 1030 Vienna, Austria;; ^b^International Institute for Applied Systems Analysis, 2361 Laxenburg, Austria;; ^c^Vienna Institute of Demography, Austrian Academy of Sciences, 1030 Vienna, Austria;; ^d^Wittgenstein Centre for Demography and Global Human Capital, International Institute for Applied Systems Analysis, Austrian Academy of Sciences and University of Vienna, 2361 Laxenburg, Austria;; ^e^Scripps Institution of Oceanography, University of California San Diego, La Jolla, CA, 92093;; ^f^Department of Social and Political Sciences, Bocconi University, 20136 Milan, Italy

**Keywords:** sustainability science, human well-being indicator, basic needs, survival, good life

## Abstract

Sustainable development (SD) as popularized by the Brundtland Commission and politically enshrined in the Sustainable Development Goals has been the explicit focus of sustainability science. While there is broad agreement that the trend of human well-being (W) over time should serve as a sustainability criterion, the literature so far has mostly addressed this in terms of its determinants rather than focusing on W itself. There is broad agreement that an indicator for W should have multiple constituents, clearly going beyond gross domestic product. Here, we propose a tailor-made indicator to serve precisely this purpose following a set of specified desiderata, including its applicability to flexibly defined subnational populations by gender, place of residence, ethnicity, and other relevant characteristics. The indicator, years of good life (YoGL), reflects the evident fact that in order to be able to enjoy any quality of life, one has to be alive and thus is primarily based on life expectancy. However, since mere survival is not considered good enough, life years are counted conditional on meeting minimum standards in two dimensions: the objective dimension of capable longevity (consisting of being out of absolute poverty and enjoying minimal levels of physical and cognitive health) and the subjective dimension of overall life satisfaction. We illustrate the calculation of this indicator for countries and subpopulations at different stages of development and with different degrees of data availability.

## Significance

Attempts at comprehensive quantitative assessments of sustainable development can focus on either determinants or constituents of long-term human well-being. While much research on determinants has relied on economic concepts of capital and inclusive wealth, here we focus on the constituents of well-being using a demographic approach. We construct a tailor-made metric based on life expectancy and indicators of objective and subjective well-being. The future trend in this metric has the potential to serve as a sustainability criterion and marks a crucial step in the endeavor to comprehensively assess sustainable development. At this stage, it is only applied to observed past and current conditions. To address sustainability, it will be combined with scenarios addressing future changes including feedback from environmental change.

Sustainability science refers to the most comprehensive scholarly effort to understand the interactions between natural and social systems in order to assess whether certain developmental pathways can be considered sustainable. This should also include the possible negative effects of environmental changes, such as climate change and biodiversity loss, on future human well-being. In this paper, we propose a tailor-made indicator to assess long-term human well-being as the ultimate end of sustainable development. This indicator, called “years of good life” (YoGL), is designed in such a way that it can be both empirically measured—which is the focus of this paper—and modeled in its long-term future trends—which will be the focus of future work.

When assessing changes over time in the well-being of certain human populations (or subpopulations, as defined, e.g., by gender, ethnicity, urban/rural place of residence, or other social groupings), one can focus on the determinants or the constituents of well-being. In sustainability science, thus far, empirical and theoretical research has placed more emphasis on studying the determinants, including environmental services (1), whereas specifying its constituents has received less systematic attention, often leaving us with nothing but the unspecific notion of “utility.” The focus on determinants has led to the concept of “inclusive wealth” (IW) which can be used to assess whether a society is on a sustainable development trajectory in terms of the productive base necessary to maintain a high standard of living in the future (2). However, empirically measuring the values and relative effects of the different capitals determining human well-being remains extremely challenging and “no current attempt to date can be said to be fully inclusive” (3).

The idea behind YoGL, on the other hand, is to study sustainability by focusing explicitly on the constituents of well-being and its change over time. In doing so, YoGL avoids several of the pitfalls by which the IW approach is plagued (3, 4). For example, rather than making contestable quantitative assessments of the relative contributions of the different determinants of well-being, the demographic approach underlying YoGL provides numerical values of human well-being directly, expressed as the average number of years of good life a person can expect to live as part of a given subpopulation under the conditions of a specified point in time. Based on the assumed universal nature of its unit of measurement—YoGL lived today in one specific population has the same meaning as YoGL lived in the future or in another population—the indicator has a time-independent meaning. This also avoids the pitfalls of specifying a rate at which to discount future well-being, which have become apparent at least since the debates around the Stern report (5). YoGL allows us to directly compare human well-being across different subpopulations and generations. Moreover, while all estimates of the different determinants of future human well-being are highly sensitive to population growth, as a measure referring to per-person well-being the derivation of YoGL is not directly affected by assumptions about the future trajectory of population size. Finally, as stressed by Dasgupta (6), the nature of determinants can change over time and across places depending on different commodities and technological regimes, whereas the constituents of well-being—as used in YoGL—are arguably shared across space and time.

In the following, we will first present the proposed design of the indicator. We will then provide a step-by-step user’s guide for empirically deriving YoGL based on the most appropriate available data source, before offering examples of how it can be calculated based on auxiliary information on populations for which the necessary data are not yet fully available. We will close with a discussion and brief outlook as to what is still needed to use this indicator for the assessment of sustainability.

## The Design of YoGL

The design of YoGL is characterized by a clear hierarchy among its constituent dimensions. First and foremost, we consider survival as the most essential prerequisite for enjoying any quality of life (QOL). When a person dies, there is no QOL left (at least not in this world). But since mere survival is typically not considered the same as QOL, in a next step we go on to define “good” years of life as those years when people are above a minimum level, both in terms of objectively observable conditions as well as subjective life satisfaction. Only if people are above critical levels in both dimensions are their life years considered as good years in the calculation of YoGL. The question of what constitutes a “good life” has been the subject of philosophical debates for millennia and we are not proposing to have found the ultimate answer. What the YoGL approach provides is the operationalization of one specific, quantifiable answer in terms of flexibly definable minimum standards.

**Fig. 1.**
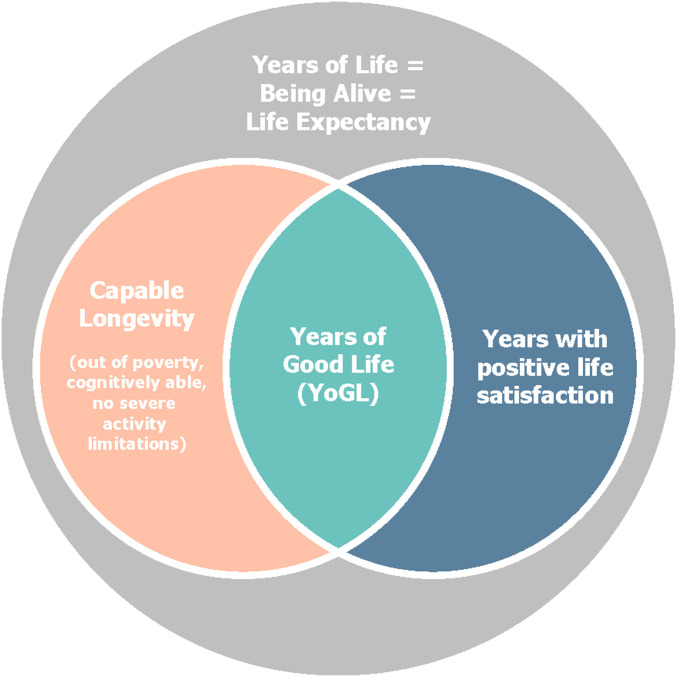
Dimensions of years of good life, a human well-being indicator.

Fig. 1 illustrates the structure and basic logic of YoGL. The big gray circle corresponds to the overall years of life a person can expect to live given the currently observed survival conditions among a specific (sub)population, as calculated through standard demographic life table methods. Although derived from individual-level information of survival or death over an observation period, life expectancy is not an individual characteristic but a characteristic of the (sub)population to which an individual belongs. YoGL is a subset of these overall years of life depicted by the green area indicating the intersection of objectively assessed capable years of life (pink circle) and years with subjective life satisfaction above a minimum level (blue area).

The vast literature on human well-being shows a clear bifurcation into considering either objective or subjective criteria for what is considered a good life. Objective measures have a longer tradition and are more numerous, whereas representative studies of subjective well-being (SWB) have only become available more recently (7). Objective and subjective measures of well-being are fundamentally different concepts. Translating and combining them into a unified framework has its complications, as has been shown by the discussions around the Easterlin paradox describing the nonlinear relationship between income and happiness (8). Yet not including both of these important aspects of human well-being risks creating an incomplete indicator which may not be acceptable to the respective opposite camp.

Therefore, in YoGL, we consider being above minimum thresholds in both SWB and objective measures of human well-being as separate necessary conditions. Being highly above the minimum on the objective indicators does not compensate for insufficient SWB and vice versa. A further advantage of introducing a purely subjective measure in an otherwise objective index is that the deeply subjective nature of SWB makes it appropriate for capturing some of the “softer,” value-related dimensions of what is considered a good life, such as living in a more egalitarian society, experiencing freedom and trust, or valuing a clean environment. For example, people might derive happiness based on different values in Western and non-Western societies (9). However, while universal agreement over such values will never be accomplished across individuals and cultures, the purely subjective statements on overall life satisfaction can be assumed to integrate all these aspects in whatever is stated as overall life satisfaction (10, 11).

The field of SWB research is rapidly expanding, with an average of 14,000 publications a year (12), and attracts more and more attention (13). There are universally recognized scales to measure SWB, notably “life satisfaction” (14, 15), “life evaluation” (16), as well as the widely used “happiness scale” and “affect balance scale” going back to Bradburn (17). All of them are used in reputable international surveys. O’Donnell and Oswald (18) suggest a possible weighting method to capture human feelings (using four items—happiness, life satisfaction, anxiety, and worthwhileness of life), yet they found that all four items are given a reasonably sized amount of importance. Furthermore, the literature suggests somewhat less stability of “happiness,” which refers to a more emotional assessment of one’s life, whereas life satisfaction yields a more reflective, and subsequently less volatile, evaluation (12, 19). For this reason, we chose to rely on overall life satisfaction rather than happiness to cover the subjective dimension in YoGL. Implicitly, one’s overall life satisfaction contains a subjective weighting of satisfaction in different life domains which contribute to overall life satisfaction.

Extensive literature reviews of well-being indicators have been published in recent years (20, 21). While much progress has been achieved in measuring SWB through surveys, the use of subjective indicators for cross-country comparisons remains contested. The factors that influence SWB vary from genetics to social conditions, with the cultural dimension playing particularly important roles when it comes to country-level heterogeneity (10, 22, 23). Challenges exist also when SWB measures are interpreted as “proxies for utilities” (13) and attempts to build indices that can be used as standard utility measures are ongoing (24, 25). Despite its recognized limits, however, at the present state of the art in SWB research, life satisfaction represents the most suitable indicator for the purpose of capturing the subjective element of YoGL. Indeed, thanks to a fairly standardized question now routinely asked in many international surveys, life satisfaction can be assessed across a large number of countries in the world. Beyond pragmatism, it is also important to stress that life satisfaction is not used here as a continuous metric that is assumed to be valid everywhere in the world. Rather, YoGL incorporates the SWB dimension the same way it incorporates objectively measurable capabilities, namely by defining a minimum threshold below which the living conditions of the respondents can be interpreted as a “cry for help,” irrespective of country or survey period. This approach minimizes the comparability concerns associated with the interpretation of SWB measures.

Independent of the subjective evaluation of life, there are objectively assessable criteria for what constitutes a good life. Desai et al. (26) identify 1) basic health, 2) basic material subsistence, as well as 3) cognitive functioning as the three “basic capabilities” that jointly determine a person’s “freedom” to achieve well-being. This general approach has later been translated prominently into the Human Development Index, whose three components (health, income, and education) directly reflect the three aspects of capability. Desai et al. also suggest combining these three dimensions with longevity into an indicator called “capable longevity.” YoGL operationalizes this general idea.

Ideally, capable longevity should be measured through objectively assessed characteristics, for example by using tested health and measures of cognitive functioning instead of simply asking individuals about their abilities. It is well-established that self-assessed physical and cognitive health measures are substantially biased in describing differences between countries, age groups, and educational groups (27, 28). We thus propose using the following measures to derive YoGL:

1) Being out of absolute poverty in high-income countries can be assessed based on household consumption or income data. In low- and middle-income countries, where such data are typically difficult to come by, household poverty status has to be assessed indirectly, for example, through the presence of long-lived consumer durables, such as a flush toilet or a solid floor in the house, the availability of which indicates material well-being as independent of culture or time period as possible.
2) Having no severe activity limitation should be assessed with respect to objectively measurable difficulties in important routine activities. We use the broadly available indicator based on testing the difficulty in rising from a chair (29), which can be objectively verified and need not rely on self-assessment.
3) Being cognitively able to function could be assessed through tested basic numeracy, memory, or literacy, with the latter only being meaningful in societies where everybody had the chance to learn to read early in life (30).

These three objective indicators are by no means entirely time- and culture-invariant, but they enable us to make a first numerical evaluation of capable longevity. We chose these indicators out of pragmatism since more consistent indicators are not yet available for most subpopulations. However, the hope is that future surveys will routinely include these measures.

YoGL shares the idea that length of life should be a necessary, but not sufficient, dimension of aggregate-level QOL with a group of other indicators [including, e.g., healthy life expectancy (31, 32), the human life indicator (33), happy life expectancy (34), or the quality-adjusted life year-based York indicator (35, 36)]. Different from these indicators, however: 1) YoGL relies on both objective and subjective indicators of QOL, capturing both individual life satisfaction and important objectively measurable dimensions of human well-being such as poverty and mental and physical health; 2) it sets minimum standards for the objective and the subjective dimensions, thus defining a discontinuous relation between the QOL dimensions and length of life; and 3) it builds on information that is available in standard surveys and is thus implementable and comparable across different countries. This last advantage of YoGL will be illustrated in the next section, where Fig. 1 will be operationalized using different data across countries of Europe and the world.

## Application and Results

### YoGL with Complete Empirical Data.

We first illustrate the application of YoGL using data from the 2013 Survey of Health, Ageing and Retirement in Europe (SHARE) which provides high-quality microlevel information on health, well-being, and socioeconomic characteristics for the population 50+. The survey is particularly well suited to analyze YoGL (at age 50), because it is ex ante harmonized across all participating countries and includes all four variables needed to compute the indicator. We base our calculations on wave 5 (2013), because it is the most recent wave including the chair stand test that is used to operationalize physical health. This wave provides the variables needed for the derivation of YoGL for 63,066 individuals aged 50 and older from 14 European countries.*

As mentioned above, a year is only counted as a good year if individuals are simultaneously 1) out of poverty, 2) free from cognitive limitations, and 3) free from physical limitations, and 4) report being generally satisfied with their lives. The calculation of YoGL requires us to set cutoffs for these four elements. Most thresholds are based on the literature and we provide sensitivity analyses whenever the literature does not give us clear guidance on where to draw the line. To make our results reproducible, we provide the codes for both the main results and the sensitivity analyses online.

#### Being out of poverty.

The threshold for being out of poverty is based on the World Bank poverty line for upper-middle income countries of US$5.50 purchasing power parity (PPP) per day (37). This cutoff corresponds to newly estimated international poverty lines from comparable national thresholds (38). In particular, poverty is assessed based on equivalized disposable household income in international dollars. Anyone having less than US$5.50 PPP per day is considered poor.

#### Being free from cognitive limitations.

SHARE includes several tests of cognitive ability. The main results presented below are based on a well-established numeracy test, for which survey participants have to answer five questions, such as “If the chance of getting a disease is 10 percent, how many people out of 1,000 […] would be expected to get the disease?” Individuals are considered free from cognitive limitations if they answer two or more questions correctly. To test the sensitivity of YoGL with respect to this assumption, 1) the cutoff is alternatively set at three or more correct answers and 2) cognition is assessed based on a memory test (rather than basic numeracy), for which survey participants have to recall a list of 10 words. Following the literature (28, 39, 40), individuals are considered free from cognitive limitations if they recall four or more words.

#### Being free from physical limitations.

Physical health is assessed based on a chair stand test, for which respondents are asked to rise from a chair without using their arms, after confirming that they felt safe to do so (see, e.g., ref. 28). The test produces a binary outcome and thus setting a cutoff to dichotomize the variable is not necessary. Respondents who did not feel safe to do the test or were unable to rise from the chair without using their arms were considered to have physical limitations. For robustness analyses, individuals that had to use their arms to stand up from the chair were also considered free from physical limitations.

#### Having positive life satisfaction.

Life satisfaction is assessed via a standard 10-step Likert scale based on the question “On a scale from 0 to 10 where 0 means completely dissatisfied and 10 means completely satisfied, how satisfied are you with your life?” Individuals are considered to have positive life satisfaction if they rate their life satisfaction to be larger than 4. In our sensitivity analysis, we set thresholds to larger than 3 as well as larger than 5.

Based on the four variables described above, a binary variable is generated that indicates whether an individual is above the critical threshold in all dimensions or not. This binary variable is then aggregated by country, gender, and 5-y age group using cross-sectional survey weights. Hence, every country–gender–age group is assigned a value between 0 and 1 that is used to divide the total number of person-years contributed by that subgroup of population into good years and bad years, thereby following the method described by Sullivan (31). Gender-specific life tables are obtained from Eurostat (41) and abridged to 5-y age intervals, using 85+ as the open-ended category. On average, 85.9% of all individuals in SHARE are simultaneously out of poverty, free from cognitive limitations, free from physical limitations, and have positive life satisfaction.

**Fig. 2.**
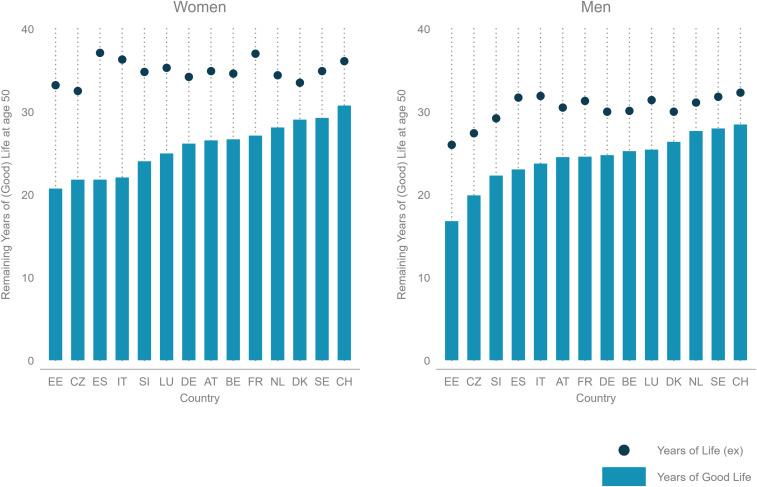
YoGL and life expectancy at age 50 for 14 European countries, 2013.

YoGL at age 50 based on SHARE data and Eurostat life tables is provided separately for men and women in Fig. 2, along with life expectancy at age 50. The countries are ranked based on YoGL separately for women and men. Results show that YoGL differs substantially between countries. Central and Eastern European countries as well as Southern European countries have the lowest values, while Northern and Western European countries have the highest values. Sensitivity analyses with respect to different cutoffs are described in *SI Appendix*, Fig. S4. While the results and country rankings are generally quite robust to modifications of the cutoff points, these analyses show that the sensitivity is greater the closer the cutoff is to the middle of the distribution. This finding supports the choice to focus primarily on the tail ends of the distributions, namely those who are without doubt in very unfavorable conditions.

As a first step toward studying the determinants of YoGL, in *SI Appendix*, we also present the indicator differentiated by the highest level of educational attainment based on SHARE data (*SI Appendix*, Fig. S1). There, strong differentials by education become visible with the better educated consistently showing higher years of good life.

### YoGL with Incomplete Data.

YoGL has been designed to be potentially applicable to any subpopulation in any country and at different points in time. While age- and gender-specific survival rates, which are needed for calculating a life table and thus total life expectancy, typically come from vital registration systems covering entire (sub)populations, the indicators used to assess the proportions of years considered as good years of life have to be derived from sample surveys. While there is a huge empirical basis of such surveys for most countries, many surveys provide only self-reported data due to resource constraints. In this subsection, therefore, we provide estimates of YoGL based on auxiliary data. We use tested measures whenever feasible and impute data or rely on proxy information when objective data are not available. We also employ regression-based prediction models to extrapolate missing information. Thus, the trends and differentials in YoGL for selected subpopulations presented in this subsection, rather than as final results, have to be treated as an exemplary demonstration to illustrate YoGL’s potential for making large-scale, international comparisons across a vast range of countries and their subpopulations over time and at different stages of development. By demonstrating this potential, we also hope to motivate more cooperation among survey takers and more systematic collection of information following the example of SHARE in the future. A full account of the data and indicators used in this section, along with the methods and the assumptions that had to be made, is given in *SI Appendix*.

The bases for the results presented in this subsection are the World Values Surveys (WVSs) along with auxiliary information from other data sources. The minimum threshold for the subjective dimension, namely life satisfaction, was again chosen to be 4 (out of a standard 10-step Likert scale). To derive the objective dimension based on WVS data, individuals are considered to be out of poverty if they are in the upper part of the respective national income distribution while reporting to be able to save part of their income. Since WVSs do not provide sufficient information on physical ability, this information is imputed based on a combination of auxiliary data sources, where individuals are considered to have severe activity limitations if they are unable to stand up from a chair or if they complete a walking test at a pace slower than 0.6 m/s. Finally, individuals’ cognitive ability is measured based on their ability to read as assessed in WVSs.

Other than that, no further modifications had to be made in comparison with the method described above for the SHARE data. As with total life expectancy, YoGL can be assessed at birth, as well as at any other age considered appropriate. Since it is problematic to assess life satisfaction for children, in our WVS-based calculations, we focus on remaining life expectancy at age 20. Table 1 shows results for 38 countries at very different stages of development and for women and men separately. As expected, the cross-country differences in YoGL at age 20 (left column) are much bigger than the differences in life expectancy (right column). While in most developed countries, women at age 20 can expect to have more than 50 y of good life left (with a record of 58 y in Sweden), women in the least developed countries can expect less than 15 y (with a record low of 10 y for women in Yemen). While life expectancy is higher for women than for men in every single country, female YoGLs turn out to be lower than male in most developing countries. This reveals a significant gender inequality in objective living conditions and subjective life satisfaction in most of these countries.

**Table 1.** YoGL at age 20 for 38 countries

Country	Female		Male	
	YoGL, y	LE, y	YoGL, y	LE, y
Sweden	58	64	55	60
The Netherlands	57	64	56	60
Germany	54	63	51	58
Chile	52	62	50	57
China	52	59	50	56
Spain	52	66	51	60
South Korea	51	65	51	58
Cyprus	50	63	50	58
Estonia	49	62	44	52
Uruguay	49	62	47	55
Ecuador	48	61	47	56
Brazil	47	60	45	53
Thailand	47	60	47	53
Colombia	46	59	44	53
Lebanon	45	62	46	58
Malaysia	43	58	44	54
Romania	43	59	42	53
Peru	42	59	46	55
Mexico	40	61	44	57
Armenia	39	58	39	52
Georgia	39	58	36	50
Turkey	39	60	45	54
Kazakhstan	38	55	36	46
Russia	35	57	33	46
Jordan	34	57	36	54
Algeria	29	59	36	57
Iraq	29	55	34	51
Ghana	28	49	31	47
South Africa	25	47	25	40
Pakistan	24	54	35	52
Haiti	23	51	23	48
India	23	54	31	51
Tunisia	23	59	32	55
Zimbabwe	22	44	25	42
Egypt	19	55	26	51
Morocco	14	59	12	57
Rwanda	11	52	12	50
Yemen	10	51	21	48

Columns: female YoGL, female life expectancy (LE) at age 20, male YoGL, and male LE at age 20; ordered by female YoGL.

**Fig. 3.**
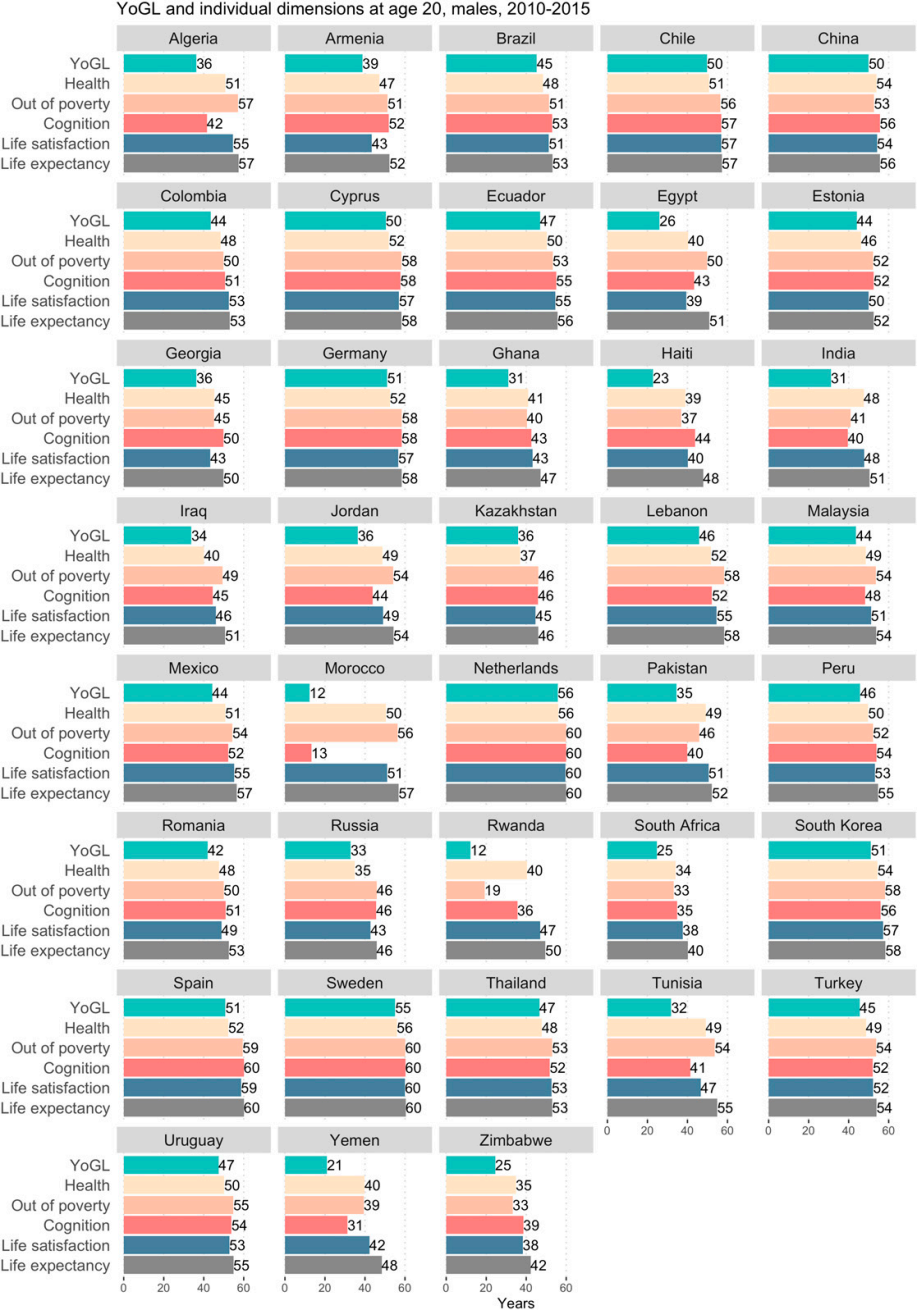
YoGL and its individual dimensions for 38 countries at age 20, males, 2010 to 2015.

Such differences naturally trigger the question of which YoGL components drive them and whether there has been at least an improving trend over time. Fig. 3 depicts the results from Table 1 for males, decomposed by the individual components of YoGL, to better understand why men are losing years of good life. The results reveal considerable differences between countries: While the reduction in “good years” due to poor health plays a proportionally larger role in highly developed countries, high levels of poverty and cognitive impairment are the main drivers of losing YoGL for men in the least developed countries. A corresponding figure depicting the decomposition results for women can be found in *SI Appendix*, Fig. S3.

**Fig. 4.**
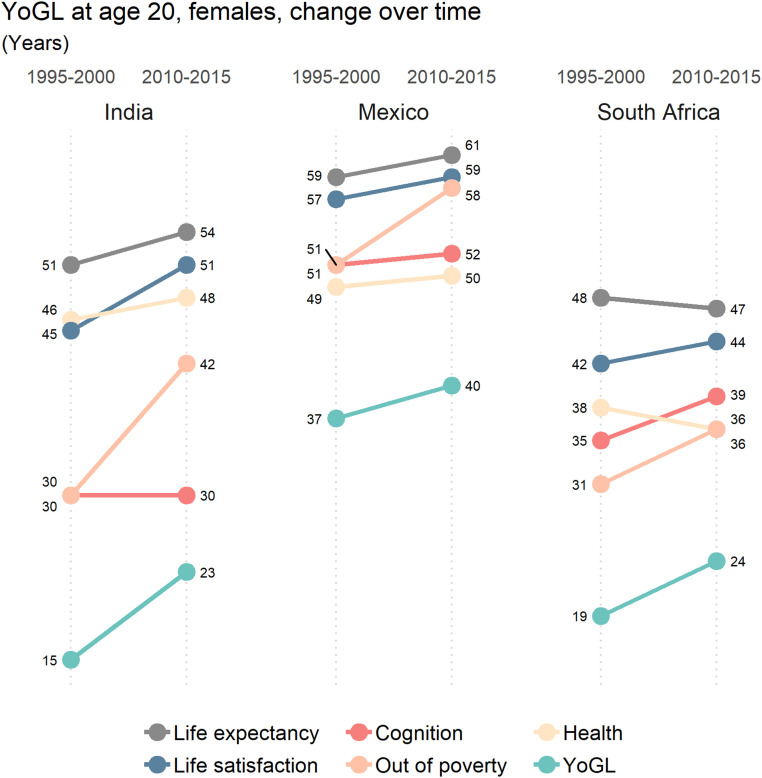
Trends in female YoGL at age 20, India, Mexico, and South Africa.

To study potential improvements over time, Fig. 4 shows the time trends in the individual components of YoGL for women and three selected countries. In India, women at age 20 in 1995 to 2000 had a total remaining life expectancy of 51 y, but only 15 of these years were assessed as being years of good life. Only 15 y later in 2010 to 2015, remaining life expectancy had increased by 3 y while YoGL increased by 8 y. The decomposition of the overall increase can be traced back primarily to reductions in absolute poverty as the main driver and to a lesser extent to improving life satisfaction and physical health. Cognition, on the other hand, has not improved over time.

South Africa displays a different pattern, with total life expectancy even declining by 1 y over the same period—presumably due to HIV/AIDS—while YoGL increased by 5 y, due to improvements in all components except for physical health. In Mexico, finally, life expectancy and YoGL increased almost in parallel, with all components showing moderate increases except being out of poverty, which showed a steep increase. With respect to gender differentials, the improvements in YoGL have been much steeper for men than for women in both India and Mexico, with little difference in South Africa. Additional results for a larger number of countries based on WVS data are given in *SI Appendix*.

## Discussion and Outlook

In this article, we propose a demography-based approach to measuring human well-being that leads to an indicator that can serve to judge long-term development trajectories with respect to their sustainability, both past and future. The years of good life indicator focuses on the changing composition of populations with regard to human characteristics that constitute the well-being of groups of people with a flexible definition of subpopulations of interest. While data availability remains an issue in the application of YoGL to most populations, as a first step our results demonstrate the feasibility of such a comprehensive indicator for a better understanding of sustainable development, where sustainability is defined in terms of changes in YoGL over the long run. More precisely, if development is to be called sustainable, YoGL should not decline over time, even when factoring in feedback from environmental and economic changes.

The proposed indicator meets several important desiderata for any metric of sustainability. They are spelled out in detail in *SI Appendix* and then applied to a list of 30 other well-being indicators proposed in the literature. These desiderata range from being applicable to subpopulations of interest to being comparable over time and across subpopulations to having a substantive interpretation in their absolute value rather than being a relative index. Also, the focus of YoGL on what can broadly be interpreted as “survival in an empowered condition” seems to come as close to near-universal acceptability as an ultimate end of human development as possible. In the context of the Global Burden of Disease Study, large empirical investigations were carried out on different continents confirming interculturally shared values in assessing health outcomes (42). However, due to the evident multitude of world views and values, there can never be fully universal agreement on ultimate ends (43). YoGL tries to avoid the need to explicitly specify certain potentially contested values by letting people judge for themselves about what is their overall life satisfaction according to their own values and their own weighting of satisfaction in different domains of life. Only if overall life satisfaction is above a minimum level are life years counted as good years. Data availability on this subjective dimension obviously sets a natural limit to how far we can go back in reconstructing YoGL for the past. However, as has been shown for the case of Finland, which has the longest demographic time series going back to 1722, there are ways to reconstruct YoGL based on certain assumptions even for the distant past (44).

Another important advantage of YoGL, especially over those indicators that can be assessed only at the national level, is its applicability to flexibly defined populations and subpopulations. In accordance with often-voiced criticism against composite well-being indicators (e.g., in ref. 45), which are not based on individuals and thus lose important information on individual-level correlations in the aggregation process, YoGL is defined in a bottom-up manner. YoGL takes individual-level information and aggregates it to the level of (sub)populations, for which life tables can be derived. As a consequence, even though YoGL is suggestive of the average years of good life an individual can expect, it is designed for assessment at the level of groups of people.

It is also important to highlight that YoGL is a member of a family of well-being indicators that are based on life expectancy. They range from only using mortality data to incorporating health aspects or other characteristics associated with well-being. Amartya Sen (ref. 46, p. 2) suggested that mortality by itself can serve as an indicator of economic success. In his words, “a higher income would be instrumentally valued. On the other hand, being able to avoid starvation, hunger and premature death is valued for its own sake.” Mathematically speaking, poverty (or any of the four minimum standards) represents a discontinuity in YoGL, but the index is a function growing monotonically with the overall living conditions of the population: Once the minimum standards are met, length of life as resulting from mortality conditions defines the final value. This reliance on life tables does not imply that crucial dimensions such as income are not relevant. Rather, they are considered only to the extent that they effectively improve the lifespan of individuals. This is consistent with the view that economic and educational conditions are “means to other ends,” this end being a good and long life (33, 46).

This paper only addresses a step in the great challenge to comprehensively estimate the “well-being production function” of sustainability science (1, 3, 47, 48), namely the choice of a metric of well-being that can be assessed across subpopulations and over time. A major remaining challenge is the specification of feedback from environmental changes onto future long-term human well-being by using scenarios for possible future trajectories of the constituents of well-being as well as the stylized modeling of population–development–environment interactions (49). Yet, constructing and empirically estimating a tailor-made well-being indicator that can serve as the dependent variable in this analysis is an indispensable first step. The presentation of such an indicator can also lead to more and better data collection including the components of YoGL in major international surveys. An indicator like YoGL also has the potential to become a broadly used currency in which the costs and benefits of certain developments and actions can be expressed, complementing assessments based on purely monetary units. For example, the social costs of carbon could potentially be assessed in terms of years of good life lost among future generations, rather than only in some dollar terms (50, 51).

## Materials and Methods

The derivation of YoGL requires five different data inputs. The essential one of them is life expectancy, typically provided by life tables. The life tables we are using to calculate YoGL by gender and over time (presented in Figs. 2, 3, and 4) are taken from Eurostat (41) as well as the latest available revision of the UN World Population Prospects (52) that covers all countries of the world. Education-specific life tables are not as widely available as the breakdown by gender, but for a small sample of European countries and for selected years, Eurostat reports remaining life expectancies in single-year steps from age 0 for three broad education groups and gender (53). These are used to derive YoGL at age 50 by education group and gender in 2013 as presented in *SI Appendix*, Fig. S1.

In addition, YoGL requires age-specific prevalence rates for its four additional constituent dimensions—poverty, physical and cognitive health, as well as life satisfaction. Most existing surveys do not yet collect all the necessary information on each of the four individual dimensions necessary to calculate YoGL. For the results presented in Fig. 2, we utilize SHARE data (54) as described in YoGL with Complete Empirical Data. For YoGL with Incomplete Data, we utilize survey data whenever feasible but add imputations and out-of-sample predictions when needed. Since the subjective dimension of YoGL is by far the most volatile one and therefore more difficult to infer, we focus on data sources that contain at least life satisfaction and try to impute the missing variables for the sample population. Accordingly, for the results presented in Table 1 as well as Figs. 3 and 4, where the goal was to make YoGL comparisons for a diverse set of countries, we chose World Values Surveys (55) as our main database and impute the missing dimensions from the Survey of Health, Ageing and Retirement in Europe (54), Study on Global Ageing and Adult Health (56), and Multi-Country Survey Study on Health and Responsiveness (57). For the computations of YoGL at age 50 for different education groups presented in *SI Appendix*, Fig. S1, we rely solely on SHARE data, which include indicators in all four YoGL dimensions, in particular tested data, but only for a very limited sample of countries. A more detailed summary of the steps taken to derive the information presented in the paper is provided in *SI Appendix*.

## Supplementary Material

Supplementary File

## Data Availability

This article is based entirely on openly accessible public data. Data reported in this article for years of good life, a well-being indicator designed to serve research on sustainability, have been deposited at https://dare.iiasa.ac.at/114/.

## Footnotes

*Israel also participated in SHARE wave 5 but is excluded from the analysis for comparability reasons.
