# Supplementary material for: Years of good life is a well-being indicator designed to serve research on sustainability
Source: Proc Natl Acad Sci U S A. 2021 Mar 15;118(12):e1907351118. doi: 10.1073/pnas.1907351118 (PMC8000580; doi:10.1073/pnas.1907351118)
Supplement: Supplementary File [file pnas.1907351118.sapp.pdf]

PNAS
www.pnas.org

Supplementary Information for

Years of Good Life (YoGL) is a wellbeing indicator designed to serve research on sustainability

Wolfgang Lutz, Erich Striessnig, Anna Dimitrova, Simone Ghislandi, Anastasia Lijadi, Claudia Reiter, Sonja Spitzer, Dilek Yildiz*

*Corresponding author: erich.striessnig@univie.ac.at

This PDF file includes:

Supplementary text
Figs. S1 to S4
Table S1
References for SI reference citations

Content

This supplementary document for "Years of Good Life (YoGL) is a wellbeing indicator designed to serve research on sustainability" provides additional information on 1) six desiderata for defining a wellbeing indicator, 2) a comparison with 31 other wellbeing indicators, 3) data and methods for the application of YoGL, and 4) sensitivity analyses for the results of this application.

1. Six desiderata for defining a wellbeing indicator that can potentially be used as sustainability criterion

The field of wellbeing indicators is currently mushrooming. On the one hand, this reflects a deep dissatisfaction with conventional indicators of wellbeing that fail at "Measuring Tomorrow" (1) in the sense that they do not account for sustainability and resilience within social and natural systems and have therefore been described as "broken compasses for policy" (2). On the other hand, there is an evident need for quantitative indicators to help us assess whether developments go into desired directions and for comparing and benchmarking such developments across populations (an overview and comparison of alternative wellbeing indicators currently available follows in the next section of this Supplementary Material). As Donella Meadows put it: "If we can't define what our ultimate ends are, how can we know whether we are approaching them"? (3). The official UN-led statistical process assessing and evaluating the implementation of the SDGs, for example, has identified 230 indicators covering the 169 targets and 17 broader goals that should be estimated and compared across all national populations. But among such a flood of indicators it is difficult to see the big picture, in particular when their relative importance, potential synergies and trade-offs remain disputed (4).

An alternative strategy is to aim at one composite metric, a wellbeing indicator W which incorporates a number of key constituents of wellbeing. Here, at least four possible approaches in terms of combining the different constituents can be distinguished:

i. One can leave the weighting of different aspects of W to the users as is done e.g. by the OECD Better Life Index (5). While being seemingly rather user-friendly, this approach leads to non-comparable values depending on individual tastes and preferences (6).

ii. One can give fixed weights to the different dimensions within W, thus already implicitly making a choice about the substitutability among components as exemplified by the UNDP Human Development Index (HDI). This approach suffers from the implicit assumptions of problematic trade-offs between sub-indices (7).

iii. One can apply a data-driven approach to select the relative weights of different dimensions within W based on e.g. the frequency by which a specific type of hardship occurs within a population (8) or the quality of the data available for each of the dimensions to be aggregated (9). While this approach seems to save researchers the trouble of applying explicit normative judgements, the lack of theoretical reasoning, implicit judgement about the choice of indicators and the serendipity of data quality in the chosen indicators makes this approach unconvincing (10).

iv. Finally, one can develop a fully integrated indicator dominated by one metric (life expectancy in the case of YoGL) plus additional conditioning factors that must all be above a minimum level. This is the case of YoGL, where the conditioning factors are reduced to one: the Boolean conjunction of the constituent dimensions, with the result that someone's years of life will

contribute to W only if the minimum standards are met *simultaneously* in all the (four) dimensions. This way, YoGL does not apply a standard weighting structure to its constituents.

As a first step towards operationalizing this approach, following the literature we specify six key desiderata that W should meet in order to serve as a wellbeing indicator whose trend over time can be used as a sustainability criterion.

(1) It should reflect widely shared values in terms of ultimate ends.

The use of W as a wellbeing indicator only makes sense as far as there is near universal agreement that it reflects a highly desirable target (3). While extremist views may contradict and individual preferences may vary to some degree, the aspiration here is to capture the single most important ultimate end that broader groups of people with very different orientations, values and cultural backgrounds would be ready to subscribe to.

Survival and the avoidance of unnecessary premature mortality, either of ourselves or people we care about, is a prime candidate for such a universally shared goal. "Being able to live to the end of a human life of normal length; not dying prematurely, or before one's life is so reduced as to be not worth living" is ranked first in Martha Nussbaum's list of "central human functional capabilities" (11). This includes altruistic behaviors that seem to contradict the universality of survival as a goal, such as a mother's willingness to sacrifice her own life to save the life of her child or a resistance fighter's determination to risk his or her own live in defense of other lives or a group's freedom from oppression. These people contribute to life expectancy and the subjective wellbeing of the group (12) under the premise expressed in the second part of Nussbaum's statement of mere survival not being considered enough for life to be called "worth living". Rather, for most people minimum standards in terms of quality of life (QOL) need to be met.

(2) It should be based on bottom-up information that can be flexibly aggregated to ***sub-populations****.*

An often-voiced criticism against composite wellbeing indicators is that they are not based on individuals and thus lose important information on correlations at the individual level in the aggregation process (13). Moreover, there is a growing recognition – not only in sustainability science – of the importance of considering heterogeneity at the sub-national level and developing local or social group-specific indicators of sustainability (14). The use of national level indicators is also problematic under the long-term perspective of sustainable development because nations come and go and may change their boundaries.

As a second requirement, therefore, it should be possible to specify W "bottom-up", i.e. based on individually measurable (or at least possible to estimate quantitatively) characteristics that can be aggregated to sub-populations. Such a focus on sub-populations, rather than nations, is essential for answering many of the important questions in sustainability science, in particular those of distribution and inequality: how does wellbeing differ by gender or by various ethnic or socio-economic groups in a population; how does it differ by urban/rural place of residence or other geographic units? The need to focus on sub-populations rules out indicators that are computed only at the national level, such as conventional GDP estimates, and it also renders the widely used HDI unfit as a candidate for W.

(3) It should be comparable over time and across sub-populations.

For the purpose of comparing the wellbeing of certain populations at two different points in time and to see whether there has been improvement or deterioration, the indicator must have a meaning in its absolute value and not be defined on a relative scale. As an example, the life expectancy component of the HDI is defined as a fraction of the maximum national life expectancy observed in any given year. Hence, when comparing this fraction for a given population at two different points in time, it is impossible to see whether survival conditions in this population actually improved and by how much. In its relative form the index can only show whether the given population improved its relative standing to the country with the highest life expectancy. Paradoxically, a country's index value can even improve without the country actually having improved in any of the HDI dimensions (15). This is why an absolute rather than a relative metric is preferred for W.

(4) It should be theory-based and reflect key constituents of wellbeing.

There seems to be broad consensus in the literature that any one dimension alone would not be sufficient for adequately capturing the ultimate end of human development or measuring human wellbeing in its multi-dimensional nature (16). Any one-dimensional indicator can be subject to similar criticism as GDP per person in terms of mis-measuring our lives (17). Even life expectancy, which has been suggested as a good and stable indicator covering and reflecting many key dimensions of wellbeing (7), would not suffice because of its focus on mere survival rather than quality of life. On the other hand, bearing in mind the possibility of complex interactions, complementarity and limited substitutability between different dimensions (18), one should be parsimonious in the number of constituents considered and choose only key dimensions with strong theoretical grounding (19, 20). Theories and conceptual frameworks should support the definition of well-being, thus informing the decisions over which dimensions to include and how to relate them with each other. In particular, the theory should highlight the weighting structure and the trade-offs implied in a specific indicator. One way of dealing with this is to set minimum standards with respect to which no compromise or compensation across dimensions could be possible at the individual level. This is the choice we made in the combination of minimum standards and Boolean conjunction of constituent dimensions in the definition of YoGL.

(5) There should be sufficient empirical information for different sub-populations and time points to be fit for serving as the dependent variable in panel regressions.

Since the purpose of W is to be estimated for many (sub-)populations at different points in time, its constituents need to be based on empirical information from survey items or other sources that are readily available. Ideally, all pieces of information necessary for the calculation of the indicator are available for the same individuals participating in a survey to satisfy desideratum #2. However, since this will not always be the case, reasonable data integration methods could be applied to obtain missing dimensions.

(6) If possible, it should have a substantive interpretation in terms of some real-life analogy rather than just being an abstract index.

An additional strength of a good indicator of wellbeing is its interpretability in terms of a real-life analogy. As Veenhoven (21) points out, the strength of GDP per person, for example, lies in its "clear substantive meaning". It is suggestive of the amount of money the average citizen has at his/her disposal to purchase goods and services. Meanwhile, life expectancy gives the number of years one can expect to live on average. The HDI, on the other hand, does not have an intuitive interpretation and gives us an abstract

number that can hardly be associated with anything tangible. Unlike the previous ones, this property of the indicator is desirable but not absolutely necessary.

YoGL as defined and discussed in the main text of this paper meets each of these desiderata and can thus be applied to operationalize the definition of sustainability, demanding that for a change in living conditions to be called sustainable, it must not lead to a decline in wellbeing for any sub-population of interest over time. The time frame that is relevant here varies by the type of intervention. Many political reforms can affect living conditions negatively in the short run, e.g. when increased taxation has thus far only led to a reduction in disposable income while the induced positive effects still need time to diffuse. This view is consistent with Laurent stating that 'measuring sustainability needs to evaluate well-being in the long run, both after the occurrence of shocks and during normal times (1).

In the following section we will discuss wellbeing indicators that have been suggested in the literature and assess whether they meet the above described desiderata.

2. Comparing 31 other wellbeing indicators to YoGL

Over the past 50 years, a large number of institutions and researchers around the world have contributed to the development of human wellbeing indicators for the purpose of supporting governments in devising meaningful policy interventions and to spur the debate on how to best raise people's quality of life in different national and cultural contexts. Many of these indicators serve as advocacy tools, educating the public on national and international differences in quality of life and leading to the expansion in research on wellbeing (22). GDP per capita continues to be by far the most prominent and widely used indicator of human wellbeing to this day, despite warnings by its inventor against its use for this purpose (23). Yet after heavy criticism of the concept (17), the majority of modern wellbeing indicators look beyond the measurement of national income and pay more attention to social and ecological dimensions of human development, including health, social capital, governance, civil liberties or environmental quality (24–26).

The desire to go beyond GDP has been highlighted by the prominent report "Mis-Measuring our Lives" where the economists Stiglitz, Sen and Fitoussi (17) discuss appropriate metrics aside from GDP per capita. In their conclusions, the authors clearly state that sustainability assessments require a well-identified dashboard of indicators. But they also stress that assessment of sustainability must be examined separately from the question of current well-being and warn against mixing or blending indicators of current well-being with indicators of sustainability that mostly will play out in the future.

While many of the more recent proposals for indicators follow the suggestion of producing a dashboard by covering indicators from a broad range of domains, they do not follow the suggestion with respect to separating the measurement of current well-being from the issue of sustainability. The Happy Planet Index (8, see also item #15 in Table S1), for instance, combines current mortality conditions in different countries – as summarized by life expectancy and data on life satisfaction – with the ecological footprint. While mortality and stated life satisfaction are measures of current wellbeing, the ecological footprint is not directly reflected in current conditions but instead measures possible impacts on future conditions, thus referring to sustainability. It therefore has a dual function which makes its direct interpretation difficult.

The conceptually clearer way – following the suggestion by Stiglitz et al. (17) – would be to have a period indicator that reflects only current conditions at every point in time and allows observing trends over

longer time periods (including possibly projections for the future) in order to follow its evolution in response to e.g. deteriorating environmental conditions. The OECD Better Life Index (item #19 in Table S1) is consistent in this respect by reflecting only current conditions (6). Through an interactive online-interface, it allows the user to choose from eleven domains, ranging from current conditions in housing and income to life satisfaction and work-live balance. These can be weighted according to the user's own assessment before aggregation of the indicator across domains.

While the above approach is internally consistent and reflecting only current conditions, the OECD Better Life Index is not designed for the analysis of sustainable development. Like many other multi-dimensional indicators, such as the Multi-Dimensional Poverty Index (28), it depends on a large number of empirical measurements. These are typically collected in surveys and can hardly be projected into the future based on a model, which is necessary for making forecasts. The simpler the indicator, the easier it is to build such a model because less assumptions need to be made and less feedbacks need to be considered. For this reason, meaningful model-based long-term scenarios exist for certain indicators, such as life expectancy and GDP per person, but it would be hard to come up with a model for forecasting the Better Life Index.

For a comprehensive literature overview of the contemporary field of wellbeing indicators see Lijadi (29). Selected examples of prominent indicators summarizing different approaches currently in use can be found in Table S1 below, ordered by year of appearance. What all of these indicators have in common is that they cover different aspects of human wellbeing, thus acknowledging the multi-dimensional nature of human wellbeing, while increasingly shifting the focus away from the dominance of the economic system to individuals and households. But as suggested by OECD, it is also important to "*reconcile the objective measures of wellbeing with those based on individual perceptions, which reflect how people actually experience and assess their life circumstances*." (30) In addition, a human wellbeing indicator useful for the study of sustainable development should be derived bottom-up to be applicable to sub-populations and focus primarily on long-term developments that can be measured in time series (31).

YoGL fulfills all these requirements and several more. It still takes material conditions into account but places them on a par with other essential dimensions of human wellbeing. The choice of the objective and subjective YoGL dimensions that are superimposed on life expectancy is based on solid theoretical foundations, considering not just economic theories of wellbeing (32–34) but also theories of subjective wellbeing (35, 36), while recognizing the important role of both the cognitive (37, 38) and physical health (39–41) dimensions of human wellbeing.

Finally, YoGL is unequivocal in not confounding means and ends of wellbeing. For example, rather than using years of schooling – which is a means to an end – as a proxy for cognitive wellbeing, YoGL measures the years a person can expect to live in a state of numeracy or functional literacy and without cognitive limitations restricting them in conducting their daily life. Schooling serves as the means of achieving numeracy and literacy, but YoGL is calculated based on the "ends", not the "means" of a good life (i.e. functional literacy, not years of schooling). This property of the indicator is crucial for using YoGL as a dependent variable in a "wellbeing production function". Indicators mixing means and ultimate ends include, for example, the Sustainable Society Index [#14], the OECD Better Life Index [#19], or the Social Progress Index [#22].

None of the existing indicators meet all of the above defined six desiderata for a fit-for-purpose wellbeing indicator for the analysis of sustainability. For example, the Human Development Index (HDI) [#4] as one

of the most widely used indicators is only compatible with desideratum #5 specified above, as the index is fit for serving as the dependent variable in panel regressions. However, the HDI uses GNI as one of the dimensions, which only exists at a national level and thus cannot be flexibly aggregated to sub-populations. Also, it is not comparable over time, since the life expectancy component of the HDI is defined as a fraction of the maximum national life expectancy observed in any given year. The HDI mixes ultimate ends (i.e. life expectancy) with means (i.e. years of education). Finally, the index is reported as an abstract index without real-life analogy.

A number of indices were developed exclusively for a specific country, such as Gross National Happiness for Bhutan [#16], the Equitable and Sustainable Well-being Index for Italy [#18], the Canadian Wellbeing Index [#20], the Thai Happiness Index [#27], the Leading Health Indicator [#30], and the Living Standard Framework Dashboard [#31]. All of them claim that the chosen dimensions were determined based on the country's specific perspective on human well-being. However, these country-specific characteristics rule out any comparisons across nations and populations and thus do not fulfill desideratum #3.

In the table below, we also report indices that meet all but the first desideratum, as they only consider one specific wellbeing dimension. Examples include Happy Life Expectancy [#8] (42), which relies solely on the subjective well-being indicator of happiness, or Literate Life Expectancy [#12] (43, 44), which uses self-declared literacy as indicator for the average number of years a person lives in the literate state. Other indices that employ life tables and are calculated by using the Sullivan method are heavily focused on health and health outcomes, e.g. Quality-adjusted Life Years (QALY) [#1] (45), calculated by estimating the years of life remaining for a person following a particular treatment or intervention and weighting each year with a quality-of-life score; Disability-adjusted Life Years (DALY) [#6] (46), calculated by the number of years a person lives free of disease and injury; and Disability-free Life Expectancy (DFLE) [#9] (47) which is the average number of years a person is expected to live free from disability.

No.	Index Name	Agency or Author(s)	Theoretical Framework	Indicators	Established and coverage	Data Source	Index Calculation	Compatibility with six criteria*
1	Quality-adjusted Life Years (initiated in 1968)	The concept is developed by Klarman et al. (48).<br>The terms QALY is first introduced by Zeckhauser & Shepard (49).	• Adjusting life expectancy based on the levels of health-related quality of life individuals are predicted to experience throughout the course of their life, or part of it.<br>• The index can be used for cost-effectiveness analysis of health care.	1. Life expectancy<br>2. Disability data<br>3. Intervention (medical, instrument/technological based support, etc.)<br>4. Duration of the disease	• QALY and DALY are currently complementing each other in assessing health outcomes. Popular in health science reports.	• Mortality rates or life expectancy from national survey<br>• Duration of disease	• QALY is measured by adjusting the life table according to the lifespan a person lives with disease.<br>• QALY gained is measured by improvement in quality of life due to some intervention to combat the disease (50).	• Criteria met: 2, 3, 4, 5, 6<br>• Only considers survival and health dimension
2	Measure of Economic Welfare (MEW) (1972)	Nordhaus & Tobin, 1972 (51)	• Alternative to crude GDP by adjusting the national output to include an assessment of the value of leisure time and the amount of unpaid work in an economy (increasing the welfare value of GDP) and the value of the environment damage caused by industrial production and consumption (reducing the welfare value of GDP)<br>• Forerunner of later attempts to create a sophisticated index of sustainable development	1. Net national product<br>2. Non-market activities and leisure time<br>3. Government final expenditures (reclassification into intermediates, consumption and net investment)<br>4. Consumer durables<br>5. Instrumental or defensive expenditures<br>6. Disamenities of urbanization	• US National Economic Accounts Data	• Calculated for the period 1929-1965 in the U.S.	• MEW = Value of GDP + Value of leisure time + Value of unpaid work + Value of environmental damage	• Criteria met: 3, 4, 5, 6<br>• Only considers economic welfare<br>• Based on national level indicators
3	Physical Quality of Life Index (PQLI) (since 1977)	Overseas Development Council, USA (34)	• Based on the claim that GDP is not sufficient to explain development.<br>• Development should be such that the physical quality of life of human beings is improving.	1. Adult literacy rate<br>2. Life Expectancy at age one<br>3. Infant Mortality Rate	• Initiated in 1977 for 150 countries	• UNESCO<br>• United Nation Population Division in the UN Department of Economic and Social Affairs (UNDESA).<br>• UNICEF	• The three indicators are combined and weighted equally.<br>• The literacy rate is a function of the per capita spending levels on education, estimated cross-sectionally.	• Criteria met: 2, 3, 5<br>• Only considers survival and literacy<br>• Literacy rate not an ultimate end<br>• Arbitrary weighting<br>• Abstract index
4	Human Development Index (HDI) (since 1990)	UNDP http://hdr.undp.org/en/content/human-development-index-hdi	• Capability Approach (Sen, 1997)	1. Life expectancy at birth<br>2. Average of mean years of schooling for adults aged >20 years, and expected year of schooling for children of school entering age	• Annual since 1990<br>• 189 countries as of 2018	Data from international and reputable organizations:<br>• UN Population Division in UNDESA<br>• UNESCO Institute for Statistics<br>• ICF Macro Demographic and Health Survey	• Equal-weighted geometric mean of three dimensions<br>• Threshold is applied to GNI, the minimum is US$100, and the maximum is US$75k per capita	• Criteria met: 5<br>• No consideration of SWB<br>• Mixes ultimate ends with means<br>• Arbitrary weighting<br>• Based on national level indicators<br>• Time inconsistent

No.	Index Name	Agency or Author(s)	Theoretical Framework	Indicators	Established and coverage	Data Source	Index Calculation	Compatibility with six criteria*
				3. Gross National Income (GNI) per capita		• UNICEF Multiple Indicator Cluster SURVEYS<br>• OECD<br>• World Bank<br>• IMF<br>• United Nations Statistics Division		• Arbitrary weighting<br>• Abstract index
5	Personal Wellbeing Index (since 1994)	International Wellbeing Group, Australian Centre on Quality of Life, Deakin University http://www.deakin.edu.au/research/acqol/iwbg/index.php	• Measuring wellbeing based on subjective global life satisfaction on the impact of community and public policy.	Overall life satisfaction in:<br>1. Standard of living<br>2. Health<br>3. Achieving in life<br>4. Relationships<br>5. Safety<br>6. Community-connectedness<br>7. Future security<br>8. Religion/spirituality (added in 2006, then become optional in 2013)	• Initiated in 1994 by Comprehensive Quality of Life for Australia<br>• In 2002, the PWI is validated as cross-cultural instrument.<br>• PWI has been adopted to more than 50 countries since 2010	• International reputable surveys as sources for individual countries	• Accumulated score from each domain (score 0-10)	• Criteria met: 2, 3, 5<br>• Only considers SWB<br>• Arbitrary weighting<br>• Abstract index
6	Disability-adjusted Life Years (since 1994)	World Health Organization (53)	• Using QALY framework in assessing health situation in populations by combining both premature death and non-fatal consequences of disease and injury.<br>• The index incorporates an age-weighting function assigning different weights to life years lived at different ages.	1. Mortality rates<br>2. Disability data (Global Burden Disease)<br>3. Disability weighting (Murray, 1994, p. 438)	• The index is popular and regularly used worldwide.	• Mortality rates and disability data from WHO	• Sum of the Years of Life Lost (YLL) due to premature mortality in the population and the Years Lost due to Disability (YLD) for people living with a health condition or its consequences:	• Criteria met: 2, 3, 5, and 6<br>• Only considers survival and health<br>• Arbitrary weighting
7	Happy Life Years (since 1996)	Erasmus Happiness Economics Research Organisation https://worlddatabaseofhappiness.eur.nl	• Assessing the quality of life in a nation requires an estimation of the total of experienced well-being, summed up as 'happiness'. Happiness is a person's overall evaluation of his/her life as-a-whole.	1. Happiness Score<br>2. Life Expectancy	• Initiated in 1996 for 50 countries	• World Database of Happiness<br>• United Nations Life Expectancy	• Multiplying life expectancy with average national happiness score	• Criteria met: 3, 4, 5, 6<br>• Only considers survival and happiness<br>• Based on national level data (average national happiness score)

No.	Index Name	Agency or Author(s)	Theoretical Framework	Indicators	Established and coverage	Data Source	Index Calculation	Compatibility with six criteria*
8	Happy Life Expectancy (1996)	Veenhoven (1996), Yang (2008), (42, 54)	• Positive and global measures of perceived quality of life at the population level by combining subjective well-being (measured by happiness) and life expectancy	1. Life expectancy (from US census)<br>2. Happiness	• First calculated in the early 1990s for 48 countries<br>• Long-term assessment (1970-2000) for the U.S. in 2009	• General Social Survey, US for happiness<br>• Life table functions by sex and race from the decennial life tables of the U.S. population from the National Vital Statistics Report	• Calculated by combining age-specific prevalence rates of subjective well-being from a large nationally representative survey and life table estimates of mortality rates and life expectancies.	• Criteria met: 2, 3, 4, 5, 6<br>• Only considers survival and SWB
9	Disability-Free Life Expectancy (since 1997)	Crimmins, Saito & Ingegneri (55)	• Healthy life-expectancy, which combines absence of disease and longevity.	1. Mortality rates to measure life expectancy<br>2. Global Activity Limitation indicator (GALI)	• The index is popular and regularly used worldwide.	• For US: mortality rates from US Vital Data; Disability prevalence data for community dwelling population from National Health Interview Survey and disability prevalence data for institutional populations from US Census.<br>• For other countries: mortality rates and life expectancy from UN, disability data from WHO, EU-SILC, etc.	• Combining age-specific mortality rates and age-specific proportion of Global Activity Limitation Indicator by employing Sullivan method.	• Criteria met: 2, 3, 4, 5, 6<br>• Only considers survival and self-reported health
10	Well-being of Nations (2001)	World Conversation Union & International Development Research Centre (IDRC), Canada https://www.idrc.ca/	• Bellagio guidelines for assessing of progress toward sustainable development at the global and national scale, including the choice and design of indicators, their interpretation and communication of the result.<br>• Quality of life is measured through the interaction between human and their environment.	Human well-being (HWI):<br>1. Health<br>2. Wealth<br>3. Knowledge & Culture<br>4. Community<br>5. Equity<br>Ecosystem well-being (EWI):<br>6. Land<br>7. Water<br>8. Air<br>9. Species & genes<br>10. Resource use<br><br>• Wellbeing Index juxtaposes HWI and EWI<br>• Wellbeing/Stress Index (WSI) shows how much human well-being each nation obtains for the amount of ecosystem stress it causes	• 183 countries in 2001	• Data from international and reputable organizations (WHO, UNESCO, FAO, UNDP, World Bank, IMF, Eurostat, GEMS, OECD, etc.)	• Equal-weighted average of HWI and EWI dimensions<br>• The Wellbeing Index is the point on the Barometer of Sustainability where the HWI and the EWI intersect<br>• The Wellbeing/Stress Index (WSI) measures the ratio of human wellbeing to ecosystem stress.<br>• Well-being of Nations index is from 0-100	• Criteria met: 5<br>• No consideration of SWB<br>• Mixes ultimate ends and means<br>• Based on national level data<br>• Not comparable over time (only one report in 2001)<br>• Arbitrary weighting<br>• Abstract index

No.	Index Name	Agency or Author(s)	Theoretical Framework	Indicators	Established and coverage	Data Source	Index Calculation	Compatibility with six criteria*
11	Index of Economic Well-being (2002)	Osberg & Sharpe, 2002 (8)	• Attempt to find plausible alternative to GDP for measuring economic well-being without ignoring effective consumption, value of accumulation, inequality, and uncertainty	Indicator is based on four dimensions of economic well-being:<br>1. Consumption<br>2. Accumulation<br>3. Income distribution<br>4. Economic security	• First calculated in 2002 for 6 OECD countries for the period 1980-1999<br>• New estimates for 14 OECD countries for the period 1980-2007 in 2009	• OECD National Accounts publication<br>• OECD Flows and Stocks of Fixed Capital<br>• IMF's International Financial Statistics Yearbook<br>• International Energy Agency<br>• LIS Database<br>• OECD Health Data<br>• UN Demographic Yearbook	• Combining the four dimensions by equal weighting (25% each) or alternative weighting (70% consumption + 1% wealth stocks per capita + 10% income distribution + 10% economic security)	• Criteria met: 3, 5<br>• Only considers economic dimension<br>• Based on national level data<br>• Arbitrary weighting<br>• Abstract index
12	Literate Life Expectancy (initiated in 1995, first calculated in 2004)	International Institute for Applied Systems Analysis (www.iiasa.ac.at) (43)	• Indicator of social development and quality of life, by measuring human development through education.<br>• The index is a real-life experience of number of years a person lives in the literate state.	1. Life expectancy<br>2. Self-declared Literacy	• First calculated for 13 world regions in 2004	• Life expectancy from 1995 Demographic Yearbook of United Nations 1997<br>• Education attainment from Statistical Yearbook of UNESCO 1998<br>• Data for Mexico from IIASA Working paper WP 96-103.	• Combining age-specific mortality rates and age-specific proportion of literate employing Sullivan method.	• Criteria met: 2, 3, 4, 5, 6<br>• Only considers survival and self-declared literacy<br>• Literacy as no ultimate end
13	Gross National Wellbeing (Since 2005)	International Institute of Management, USA	• Based on the philosophy that the ideal purpose of governments is to promote happiness.	Measuring overall satisfaction in 7 dimensions:<br>1. Mental & Emotional Wellbeing<br>2. Physical & Health Wellbeing<br>3. Work & Income Wellbeing<br>4. Social Relations Wellbeing<br>5. Economic & Retirement Wellbeing<br>6. Political & Government Wellbeing<br>7. Living Environment Wellbeing	• Initiated in 2005<br>• USA only	Online survey including four qualitative questions:<br>1. What are the top positive things in your life that make you happy?<br>2. What are the top challenges and causes of stress in your life?<br>3. What would you advise your government to increase your well-being and happiness?<br>4. What are the most influential city, state, federal or international projects? How are they impacting your well-being and happiness (positively or negatively)?	• Equally-weighted average of all dimensions.	• Criteria met: 2, 3, 5<br>• Only considers SWB<br>• Arbitrary weighting<br>• Abstract index
14	Sustainable Society Index (SSI) (since 2006)	Sustainable Society foundation of the Netherlands http://www.ssfindex.com /ssi/	• Brundtland Report on Sustainable society approach: a sustainable society is a society that meets the needs of the present generation, without compromising the ability of future generations to meet their own needs; and in which	HUMAN WELL-BEING<br>• Basic Needs<br>1. Sufficient Food<br>2. Sufficient to drink<br>3. Safe sanitation<br>• Personal Development and health<br>4. Education<br>5. Healthy Life<br>6. Gender Equality	• Bi-annual since 2006<br>• 154 countries as of 2016<br>• Excluded subjective well-being	• Data from FAO, UNESCO, World Bank, World Economic Forum, Population growth database, UNEP-WCMC, Aquastat, Global Footprint network for consumption, Renewable energy from IEA, Organic farm from FiBL, ILO, IMF World Economic Outloook	• Equal- weighted geometric means of three main dimensions<br>• SSI is from 0-10	• Criteria met: 3, 5<br>• No consideration of SWB<br>• Mixes ultimate ends and means<br>• Based on national level data (e.g. GDP, public debt, etc.)<br>• Arbitrary weighting<br>• Abstract index

No.	Index Name	Agency or Author(s)	Theoretical Framework	Indicators	Established and coverage	Data Source	Index Calculation	Compatibility with six criteria*
			each human being has the opportunity to develop itself in freedom, within a well-balanced society and in harmony with its surroundings	• Well-balanced society<br>7. Income Distribution<br>8. Population Growth<br>9. Good governance<br>ENVIRONMENTAL WELL-BEING<br>• Natural Resources<br>10. Biodiversity<br>11. Renewable water resources<br>12. Consumption<br>• Climate & Energy<br>13. Energy Use<br>14. Energy Savings<br>15. Greenhouse Gases<br>16. Renewable Energy<br>ECONOMIC WELL-BEING<br>• Transition<br>17. Organic Farm<br>18. Genuine Saving<br>19. GDP<br>20. Employment<br>21. Public debt				
15	Happy Planet Index (since 2006)	New Economics Foundation http://www.happyplanetindex.org	• General utilitarian principles: most people want to live long and fulfilling lives, and the country which is doing the best is the one that allows its citizens to do so, whilst avoiding infringing on the opportunity of future people and people in other countries to do the same.<br>• Efficiency of consumption natural resources may lead to long, happy, sustainable lives	1. Ecological Footprint<br>2. Well-being (life evaluation)<br>3. Life expectancy	• Initiated in 2006 with 178 countries<br>• 2009: 143 countries<br>• 2012: 151 countries<br>• 2016: 140 countries	• Data from Global footprint network, Gallup World Poll (life evaluation) and United Nations (life expectancy).	• HPI=Well-being x Life expectancy x Inequality of outcome / Ecological Footprint<br>• Inequality of outcome= % of life expectancy and well-being	• Criteria met: 3, 5<br>• Mixes ultimate ends and means<br>• Based on national level data<br>• Arbitrary weighting<br>• Abstract index

No.	Index Name	Agency or Author(s)	Theoretical Framework	Indicators	Established and coverage	Data Source	Index Calculation	Compatibility with six criteria*
16	Gross National Happiness (since 2008)	Centre for Bhutan Studies & Oxford University	• Measurement of collective happiness and well-being of the Bhutan population	1. Psychological well-being<br>2. Health<br>3. Time use<br>4. Education<br>5. Cultural diversity and resilience<br>6. Good governance<br>7. Community vitality<br>8. Ecological diversity and resilience<br>9. Living standards	• Since 2008<br>• Only for Bhutan<br>• no regular intervals (so far 2012 and 2015)	• Survey data by Centre Bhutan Studies	• Each dimension is composed of subjective (survey-based) and objective indicators<br>• Dimensions weigh equally but the indicators within each domain are unweighted<br>• Alkire-Foster method<br>• Three cut off 50% (unhappy), 66% (narrowly happy) and 77% (deeply happy)	• Criteria met: 2, 3, 5<br>• Mixes ultimate ends and means<br>• Arbitrary weighting<br>• Abstract index
17	Living Standards by Equivalent Incomes (2009)	Fleurbaey & Gaulier (2009) (56)	• Measure of living standards for international comparisons based on GDP per capita, corrected for international flows of income, labor, risk of unemployment, healthy life expectancy, household demography and inequalities.	1. GDP/GNI per capita<br>2. Labor<br>3. Risk of unemployment<br>4. Health<br>5. Household composition	• First calculated in 2009 for 24 OECD countries	• World Bank International Comparison Program database<br>• OECD Labor Statistics<br>• OECD-STAN<br>• OECD Revenue Statistics<br>• OECD Health Data<br>• Criminal justice statistics<br>• Luxembourg Income Study database	• Calculation is based on equivalent variation of income that would make each population indifferent between its current situation and a reference situation with respect to the non-income dimension	• Criteria met: 3, 4, 5, 6<br>• Mixes ultimate ends and means<br>• Only considers economic wellbeing and survival<br>• Based on national level indicators
18	Equitable and Sustainable Well-being / Benessere e Sostenibilita (BES) (since 2010)	Italian National Institute of Statistics https://www.istat.it/en/well-being-and-sustainability/	• Measurement of the progress of society not only from an economic, but also from a social and environmental view.	6. Health<br>7. Education and Training<br>8. Work and life balance<br>9. Economic Well-being<br>10. Social relationships<br>11. Politics and institutions<br>12. Safety<br>13. Subjective well-being<br>14. Landscape and cultural heritage<br>15. Environment<br>16. Innovation, research and creativity<br>17. Quality of services	• Launched in 2010, only for Italy<br>• Inclusion of BES Annual report for economic planning as of 2016.	• Data from Institute of Nazionale Statistica, Italy (Istat), National Institute for the Educational Evaluation of Instruction and Training, Evaluation Service, EU-SILC, Bank of Italy, Ministry of Interior, Urban Water Census, Survey on urban environmental data, Ministry of Environment for Land and Sea, Hydrological instability in Italy: hazard and risk indicators, Ministry of Health on health information system, Ministry of Economic on development.	• Each indicator is given a score from 0-10, so maximum BES is 120	• Criteria met: 2, 3, 5<br>• Mixes ultimate ends and means<br>• Arbitrary weighting<br>• Abstract index

No.	Index Name	Agency or Author(s)	Theoretical Framework	Indicators	Established and coverage	Data Source	Index Calculation	Compatibility with six criteria*
19	Better Life Index (since 2011)	OECD http://www.oecdbetterlifeindex.org/	• Stiglitz & Sen report (2010)<br>• Measurement of Economic Performance and Social Progress<br>• Sen's capability approach	MATERIAL CONDITON<br>1. Housing<br>2. Income<br>3. Job<br>QUALITY OF LIVE<br>4. Education<br>5. Health<br>6. Environment<br>7. Community<br>8. Civic engagement<br>9. Safety<br>10. Work life balance<br>11. Life satisfaction	• Bi-annual since 2011<br>• 2016: 36 countries<br>• 2017: 41 countries	• Data collected by OECD and from international organizations (EU-SILC, National Statistical Office, Gallup World Data on life evaluation)	• Equally-weighted arithmetic mean of all eleven dimensions	• Criteria met: 2, 3, 5<br>• Mixes ultimate ends and means<br>• Arbitrary weighting<br>• Abstract index
20	Canadian Well-being Index (since 2011)	Atkinson Charitable Foundation University of Waterloo, Canada https://uwaterloo.ca/canadian-index-wellbeing/	• Alternative ways to promote higher quality of life	1. Community vitality<br>• Social relationships<br>• Social norms and values<br>2. Democratic Engagement<br>3. Education<br>4. Environment<br>5. Healthy populations<br>6. Leisure and culture<br>7. Living standards<br>8. Time Use	• Launched in 2011<br>• Annual report<br>• Only for Canada	• Data from Canadian social survey and other Canadian national agencies (Statistic Canada Surveys, General Social Survey, Canadian Community Health Survey, Labour Force Survey, Canadian Survey of Giving Volunteering and Participating, Canadian Election Surveys, Environment Canada, WWF living Planet index, Canadian Centre for Economic Analysis)	• Combining the scores for each domain over time to monitor overall increases or decreases in the wellbeing<br>• Human well-being is defined as the presence of the highest possible quality of life in 8 dimensions	• Criteria met: 2, 3, 5<br>• No consideration of SWB<br>• Mixes ultimate ends and means<br>• Arbitrary weighting<br>• Abstract index
21	World Happiness Report (since 2012)	United Nations Sustainable Development Solutions Network	• Based on a cross-disciplinary approach for research on well-being to include a wide-range of fields including economics, business, psychology, sociology, political science, and education.	1. Log of GDP<br>2. Healthy years of life expectancy<br>3. Social Support<br>4. Freedom to make life choice<br>5. Generosity<br>6. Perception on the corruption<br>7. Life satisfaction (life evaluation, positive and negative affect)	• Published annually (first report in 2012)<br>• 156 countries as of 2019 (including 117 countries by happiness level of the immigrants)	• Gallup World Poll<br>• WHO HALE	• Each variable measured reveals a populated-weighted average score on a scale running from 0 to 10 that is tracked over time and compared against other countries.	• Criteria met: 3, 5<br>• Mixes ultimate ends and means<br>• Based on national level data (GDP)<br>• Arbitrary weighting<br>• Abstract index
22	Social Progress Index (Since 2013)	Social Progress imperative – USA https://www.socialprogressindex.com/	• Social progress as the capacity of a society to meet the basic human needs of its citizens, establish the building	BASIC HUMAN NEEDS<br>1. Nutrition and basic medical care<br>2. Water and sanitation<br>3. Shelter	• Annually since 2013<br>• 128 countries as of 2017	• Data from international and reputable organizations (FAO, WHO, Gallup World Poll, UNESCO, UNICEF,	• Average of three dimensions - for each dimension, average of the components	• Criteria met: 2, 3, 5<br>• Mixes ultimate ends and means<br>• Arbitrary weighting<br>• Abstract index

No.	Index Name	Agency or Author(s)	Theoretical Framework	Indicators	Established and coverage	Data Source	Index Calculation	Compatibility with six criteria*
			blocks that allow citizens and communities to enhance and sustain the quality of their lives, and create the conditions for all individuals to reach their full potential.	4. Personal Safety<br>FOUNDATIONS OF WELL-BEING<br>5. Access to Basic Knowledge<br>6. Access to Information and Communications<br>7. Health and Wellness<br>8. Environmental Quality<br>OPPORTUNITY<br>9. Personal Rights<br>10. Personal freedom and Choice<br>11. Tolerance and Inclusion<br>12. Access to Advanced Education		World Economic Forum, OECD, etc.)	• SPI is average of the score from 1-100 of the 3 dimensions (Basic Human needs, Foundations of well-being and Opportunity).	
23	Gallup-Healthways Well-being Index (Since 2014)	Gallup and Healthways https://www.gallup.com/topic/WELL_BEING_INDEX.aspx	• Measurement of global well-being across five elements to provide information for policy makers on interventions and development investments to create meaningful change.	1. Physical well-being<br>2. Financial well-being<br>3. Social wellbeing<br>4. Community well-being<br>5. Purpose well-being	• Initiated in 2012 for worldwide measurement<br>• First report in 2014 for 145 countries	• Gallup World Poll data	• The Global Well-Being Index ranks countries based on the percentage of the population that is thriving in at least 3 out of 5 elements of well-being.	• Criteria met: 2, 3, 5, 6<br>• Only considers SWB<br>• Mixes ultimate ends and means<br>• Arbitrary weighting
24	Multi-dimensional Living Standards (2016)	OECD initiative on inclusive growth (Boarini et al., 2016) (57)	• Welfare measure based on preferences<br>• Builds on the equivalent income approach to develop an indicator of Multidimensional Living Standards (MDLS) that combines monetary (income) and non-monetary (health and jobs) benefits from economic growth and aggregates them across individuals with different characteristics	1. Household income (income-based living standards)<br>2. Longevity<br>3. Employment	• Fist calculated in 2016 for 26 OECD countries	• OECD Income distribution database<br>• OECD Annual National Accounts<br>• OECD Health Database<br>• Gallup World Poll<br>• European Social Survey<br>• European Value Survey<br>• European Quality of Life Survey<br>• Eurobarometer<br>• World Value Survey	• Distribution-adjusted welfare measure that aggregates income, employment and longevity weighted by preferences of people<br>• Additional life satisfaction regressions to capture the full welfare losses of unemployment with a calibration approach to capture the value of longevity	• Criteria met: 2, 3, 4, 5, 6<br>• Mixes ultimate ends and means
25	Composite Global Well-Being Index (Since 2016)	American University of Beirut, Lebanon	• Well-being is including both the material and other aspects of an individual's quality of life	4. Safety and security<br>5. Health<br>6. Education<br>7. Housing<br>8. Environment and Living Space	• First report in 2016<br>• Covers 124 countries as of 2016	• International databases (e.g. ILO, WDI, GPI, WHO-GBD, UNODC, UNHCR, Gallup World Poll, etc.)	• Unweighted arithmetic mean of all 10 dimensions<br>• 5-year average was calculated for most flow indicators	• Criteria met: 2, 3, 5<br>• Mixes ultimate ends and means<br>• Arbitrary weighting<br>• Abstract index

No.	Index Name	Agency or Author(s)	Theoretical Framework	Indicators	Established and coverage	Data Source	Index Calculation	Compatibility with six criteria*
				9. Employment<br>10. Income<br>11. Life Satisfaction<br>12. Community and Social Life<br>13. Civic Engagement				
26	Economic well-being (2016)	Jones & Klenow, 2016 (58)	• Summary statistic for the economic well-being of people in a country by incorporating consumption, leisure, mortality, and inequality,	1. Income<br>2. Consumption<br>3. Leisure<br>4. Consumption inequality<br>5. Leisure inequality<br>6. Age-specific mortality	• First calculated in 2016 for 13 countries using detailed micro data and for a broad range of countries using multi-country datasets	• Household survey data<br>• Penn World Table<br>• UNU-WIDER World Income Inequality Database<br>• World Bank's HNPStats database	• Calculation of consumption-equivalent welfare for a given specification of preferences using data on consumption, leisure, consumption inequality, leisure inequality, and mortality by age.	• Criteria met: 3, 5, 6<br>• Only considers economic well-being and survival<br>• Based on national level data<br>• Abstract index
27	Thai Happiness Index (Since 2018)	National Institute of Development Administration http://thaihappinessindex.com	• Measurement of well-being beyond economic conditions;<br>• Greater happiness for people can be achieved by policies that aim to promote good family relationships, high community quality and work life quality.	7. Qualities of Life<br>a. Health<br>b. Family<br>c. Work-life<br>d. Community<br>e. Household economy<br>f. Living environment<br>8. Philosophy of living<br>a. Sufficiency economy<br>b. Spirituality<br>c. Citizenship<br>9. Governance Society<br>a. Basic Right<br>b. Effectiveness of Public Administration<br>10. Living Standard<br>a. Household income<br>b. Housing<br>c. Education<br>11. Subjective well-being<br>a. Happiness Level<br>b. Life satisfaction level	• Initiated in 2018 for Thailand only	• Survey by National Institute of Development Administration	• Different weights are assigned to each indicator: 30% Qualities of Life, 12% Philosophy of living, 8% Governance Society, 20% Living Standard and 30% Subjective well-being<br>• Alkire-Foster method	• Criteria met: 2, 3, 5<br>• Mixes ultimate ends and means<br>• Arbitrary weighting<br>• Abstract index
28	Human Life Indicator (HLI) (Since 2019)	International Institute for Applied Systems Analysis (IIASA) www.iiasa.ac.at	• HLI measures development in human life based on solely health component, which is the life span data that	1. Life expectancy<br>2. Infant and child mortality rate	• First report in 2019, (212 countries)	• Life expectancy and infant and child mortality rates from United Nations Data 2010-2015	• Geometric average of life spans. Life spans are a modification of life expectancy, which arithmetically accounts for the	• Criteria met: 2, 3, 4, 5, 6<br>• Only considers survival and health dimension

No.	Index Name	Agency or Author(s)	Theoretical Framework	Indicators	Established and coverage	Data Source	Index Calculation	Compatibility with six criteria*
			allow comparison across time.<br>• Mortality rates are highly correlated with education and income				averages of the distribution of ages at death.<br>• Within the same life expectancy at birth, a sub-population with lower mortality rate would be expected to have higher HLI.	
29	Years of Good Life Based on Income and Health	Coockson et al, 2016 (CHE working paper 132, York University)	• QALY used as measure of quantity and health related quality of life<br>• Income interacted non linearly<br>• Objective: improve Cost Benefit Analysis for health interventions	1. QALYs<br>2. Income	• Only one WP	• Simulations calibrated with UK focused parameters	• Linear combination of additive and multiplicative utility function for QALY and the utility of consumption<br>• Weights given by a consumption-health interaction parameter	• Criteria met: 2 and 4<br>• Does not consider objective health and minimum standard applies only to income<br>• Difficult to apply, data not available at population level for most countries
30	Leading Health Indicators (LHIs) for Healthy People 2030	National Academies, of Science, Engineering and Medicine Committee (mandate from the U.S. Department of Health and Human Services – for Healthy People 2030) (59)	Healthy People 2030 framework:<br>a. incorporate appropriate health education components into the society, especially into all aspects of education and health;<br>b. increase the application and use of health knowledge, skills, and practices by the general population in its patterns of daily living;<br>c. establish systematic processes for the exploration, development, demonstration, and evaluation of innovative health promotion concepts.<br>Criteria for HP 2030 objectives should be measurable by the data cutoff in mid-2019, address issues of national	5. Life expectancy<br>6. Child Health<br>7. Child health and well-being<br>8. Self-rated health<br>9. Well-being (Cantril's scale)<br>10. Disability<br>11. Mental Disability<br>12. Substance Use<br>13. Unintentional injury deaths<br>14. All cancer deaths<br>15. Suicide<br>16. Firearm-related mortality<br>17. Maternal mortality rate<br>18. Mental Health<br>19. Oral health access<br>20. Reproductive health care services<br>21. HIV incidence<br>22. Tobacco<br>23. Obesity<br>24. Alcohol use<br>25. Immunization<br>26. Hypertension rate	• Developed for U.S.A.<br>• Updated version from Healthy People 2020<br>• Soon to launch the first report	• The U.S. Department of Health and Human Services<br>• Life evaluation (Gallup Poll)	• Equally weighted arithmetic means of all 34 dimensions	• Criteria met: 2, 5<br>• Mixes ultimate ends and means<br>• Arbitrary weighting<br>• Abstract index

No.	Index Name	Agency or Author(s)	Theoretical Framework	Indicators	Established and coverage	Data Source	Index Calculation	Compatibility with six criteria*
			importance, evidence base, and address social determinants of health equity and disparities.	27. Ambulatory Care Sensitive Conditions<br>28. Medical insurance coverage<br>29. Affordable housing<br>30. Environment Quality Index<br>31. Environment – the Heat Vulnerability Index<br>32. Education<br>33. Poverty<br>34. Food security<br>35. Civic Engagement<br>36. Social environment – Neighborhood Disinvestment Index<br>37. Social environment – Index of Dissimilarity<br>38. Social environment – Isolation Index				
31	Living Standard Framework (LSF) Dashboard (60)	The New Zealand Treasury	The LSF is broadly based on the OECD wellbeing framework, which reflects elements of the capabilities approach to wellbeing.<br><br>The LSF address factors that can expand people's choices and opportunities to live the lives they value – including health, education and income.	1. Civic engagement<br>2. Cultural identity<br>3. Environment<br>4. Health<br>5. Housing<br>6. Income and consumption<br>7. Jobs and earnings<br>8. Knowledge and skills<br>9. Safety<br>10. Social connections<br>11. Subjective wellbeing<br>12. Time use	• Developed for New Zealand only with the inclusion of cultural identity<br>• First launched in 2018<br>• Published annually	• A variety of sources including The New Zealand General Social Survey, OECD, the Ministry of Education, the Ministry for the Environment, the Ministry of Health and the Ministry of Justice.	• Equally weighted arithmetic means of all 12 dimensions	• Criteria met: 2, 5<br>• Mixes ultimate ends and means<br>• Arbitrary weighting<br>• Abstract index

Table S1. Comparison and compatibility of selected human wellbeing indices (listed according to the year of establishment) to the six pre-defined desiderata.

Note: * The six desiderata for a fit-for-purpose wellbeing indicator are: (1) The indicator should reflect widely shared values in terms of ultimate ends; (2) The indicator should be based on bottom-up information that can be flexibly aggregated to sub-population; (3) The indicator needs to be comparable over time and across sub-populations; (4) The indicator should be theory-based and reflect key constituents of wellbeing; (5) There should be sufficient empirical information for different sub-populations and time points to be fit for serving as the dependent variable in panel regressions; and (6) If possible, the indicator should have a substantive interpretation in terms of some real life analogy rather than just being an abstract index.

3. Data and Methods

To exemplify the application of YoGL, we accompany the conceptual part of the paper with results demonstrating the indicator's potential to compare wellbeing between countries, sub-populations and over time. In this section, we describe how YoGL is approximated in view of limited data availability. Despite our efforts to collect and carefully impute harmonized individual-level data for a large group of countries over time, the empirical results demonstrating the application of YoGL still have to be treated as an exemplary illustration and thus need to be interpreted with caution. We hope that future data availability will allow scholars and policy makers to untap the full potential of YoGL.

According to desideratum #2 above, for YoGL to serve as an indicator of sustainable wellbeing, it needs to be computed "bottom-up", i.e. from individual-level survey data. This represents one of the major advantages of YoGL and allows for the indicator to be compared across countries but also different sub-populations. Unfortunately, most existing surveys do not yet collect all the necessary information on each of the four individual dimensions necessary to calculate YoGL. We thus utilize survey data whenever feasible but add imputations and out-of-sample predictions when needed.

The first step in deriving YoGL is to identify a database that contains information for a large enough number of individual observations from different countries, years and sub-populations in harmonized form. The ideal database for this purpose is the Survey of Health, Ageing and Retirement in Europe (SHARE) (61), which includes indicators of all four individual characteristics needed for YoGL based on tested data. This is what is used for the computations of YoGL at age 50 presented in Figure 2 of the main text. However, SHARE is available only for a very limited sample of countries. For the results presented in Table 1 and Figure 3 and 4 of the manuscript, where the goal was to make YoGL comparisons for a diverse set of countries and over time, we had to find an alternative data source. Since the subjective dimension of YoGL is by far the most volatile one and therefore more difficult to infer, we had to find an alternative data source that contains life satisfaction for a large number of countries, to then impute the missing variables for the sample population. The best available source for that purpose is the World Values Survey (WVS) (62). The missing dimensions are imputed from SHARE (61), the Study of Global Ageing and Adult Health (SAGE) (63) and the Multi-Country Survey Study on Health and Responsiveness (MCSS) (64), respectively.

An alternative and simpler solution to compute YoGL would be to calculate age-specific prevalence rates of each of the four dimensions using different surveys and to apply those as weights to the number of person-years lived in each age group within a population. However, that would ignore important correlations that exist between the different dimensions at the individual level. These correlations can change over time, just as much as they can vary over the individual life course, e.g. as people's income situation improves with age, their life satisfaction may deteriorate because of increased stress and responsibility.

3.1 Survival

As specified in the main text, the calculation of YoGL requires information on several different dimensions of wellbeing. The essential one of those is survival, which is conventionally described in demography by life tables. Life tables are derived from individual death records which – as a minimum requirement for the computation of life tables – have to specify age at death. As survival conditions tend to differ quite heavily by gender, life tables are often reported separately for men and women within a population. The

life tables we are using to calculate YoGL by gender and over time are taken from Eurostat (65) (Figure 1) or the latest available revision of the UN World Population Prospects (66) that cover all countries of the world (Table 1 and Figure 3 and 4 in the main text).

Another important determining factor for differences in survival is education. While the pattern of highly educated sub-populations outliving less educated ones can be assumed to be almost universal, the extent of the education advantage can vary widely across countries and over time (67, 68). Education-specific life tables are not as widely available as the breakdown by gender, but for a small sample of European countries and for selected years, Eurostat (69) reports remaining life expectancies in single year steps from age zero for three broad education groups and by gender. These are used to derive YoGL at age 50 by education-group and gender presented in Figure S1.

3.2 Capable Longevity

As mere survival is not enough, YoGL incorporates four additional dimensions of wellbeing, three of which are combined under the name of "capable longevity". The dimensions that are chosen to calculate capable longevity are (1) being out of poverty, (2) being free from cognitive and (3) physical activity limitations. Only when a respondent scores above a certain threshold on all three of these dimensions, while being satisfied with their life (see 3.3 below), the respondent will be counted as contributing a person-year to the total number of good years lived within the population in that year.

Ideally, all three dimensions of capable longevity are to be taken from objective assessments of people's income situation, cognitive and physical health, respectively. However, apart from SHARE, no individual cross-national survey fulfills this strict requirement for the accurate computation of YoGL. Therefore, when objectively assessed information on the three dimensions of capable longevity is missing, we use different methods of imputation and inference at the individual respondent level.

3.2.1 Being out of poverty

Objective information on people's income situation and/or material conditions is particularly hard to come by and often needs to be inferred from proxy information, such as the availability of amenities and large consumer durables in the household. However, for reasons explained above, YoGL needs to be computed at the individual level rather than the household level. For results based on SHARE (see Figure 2 in the main text and Figure S1 in the SI), the poverty dimension of YoGL is thus covered by the survey item "total household income", which is obtained by aggregating all single income components at the household level. Individual level data is then derived by applying an equivalence scale, i.e. assigning weights to each member of the household. In particular, the square root scale is employed, which divides the total household income by the square root of the household size. This scale is used in recent OECD publications (70, 71). Finally, the equivalized household income is converted to international dollars per day, using purchasing power parity (PPP) conversion rates, and then compared to the World Bank (WB) poverty line for upper-middle income countries ($5.50/day). Individuals that fall below the WB poverty rate are classified as being poor.

In WVS, which is used for Figures 3 and 4 and Table 1 in the main text, as well as for Figure S3 in the SI, the situation is more complex, since objectively assessed information on respondents' material living conditions is not available. Thus, we observe within-country variation of poverty based on self-assessed data and weight this information so that the between-country variation of poverty matches poverty information provided by the WB (72). The within-country variation of poverty is based on two subjectively-assessed survey items: (1) people's self-declared position within their national income distribution ("On

this card is an income scale on which 1 indicates the lowest income group and 10 the highest income group in your country. We would like to know in what group your household is. Please, specify the appropriate number, counting all wages, salaries, pensions and other incomes that come in."), and (2) people's self-declared saving behavior ("During the past year, did your family save money, just get by, spent some savings or spent savings and borrowed money"). We specify that all individuals who report a higher position on the national income scale than the second step and who are members of households that were able to save money during the past year are out of poverty.

The self-reported poverty information might be subject to bias, for example, when individuals do not know about the income distribution in their country or their position in that distribution. Moreover, when asked about their income/poverty status, individuals compare themselves with their peers, e.g. with individuals from their own country. Thus, on average, individuals from high income countries "overestimate" their poverty and individuals from low income countries "underestimate" their poverty. We account for this bias by adjusting the self-reported country-mean derived from WVS to the country mean reported by the WB (72). While this indicator does not meet all necessary characteristics of capable longevity presented in the main text, it can serve as a reliable approximation of poverty allowing us to exemplify the computation and utilization of YoGL.

3.2.2 Being free from cognitive limitations

Results presented in Figure 2 of the main text are based on SHARE. The survey provides a range of cognitive indicators, including basic functional literacy, numeracy, and word recall. We conducted sensitivity analyses of how YoGL changes when either of those are used to derive the cognitive dimension. Figure S2 compares prevalence of cognitive fitness according to two different items in the SHARE survey. As these measures are heavily correlated across age groups, our choice of which one we use to calculate YoGL does not affect the results strongly. The results presented in Figure 2 of the main text are based on a numeracy test, consisting of five simple numerical questions. For example, the respondent is asked: "*One hundred minus 7 equals what?"*. Respondents who answered two or more questions correctly are classified as cognitively fit.

Unfortunately, tested information on cognition is not available in WVS, which is why to obtain the results presented in Table 1 and Figures 3 and 4 of the main text as well as in Figure S3 of the SI we have to rely on proxy information. In particular, we utilize the assessments of the interviewers from wave 6 of the WVS on whether the respondent was able to read the questions on their own during the survey. We cross-checked the resulting country-specific age distributions of literacy against data that is available from national level literacy surveys and find them to be fairly similar. Since this proxy of cognition is not available in earlier waves of WVS, we prepare out-of-sample predictions for the other waves employing country-specific binary logit models. Age, education and gender are available throughout all waves of the survey, they have the strongest predictive power and are therefore used as predictor variables.

3.2.3 Being free from physical limitations

Another requirement for a person-year to be counted as a good year of life in YoGL is for this year to be spent free from physical activity limitations. While this does not imply that a physically impaired person cannot live many good and meaningful years of life, especially if equipped with alternative means to compensate for their physical limitations, as exemplified by the late Stephen Hawking, the majority of people living with severe activity limitations today still suffer from a lack of participation possibilities, be

it for reasons of discrimination or for lack of barrier-free infrastructure. Moreover, we are merely using conventional indicators of physical activity limitations to study the age pattern in health decline as one important element of human wellbeing, as it is common practice and widely accepted in the field of epidemiology.

In SHARE we can choose between several different measures of physical limitation, such as the ability to stand up from a chair, walking speed and grip strength, but not all measures are available in each wave. While grip strength has repeatedly been proven to correlate with subsequent adverse health outcomes to predict physical disability decades later, chair stand reflects a person's current health status (73). Since current health status is what we are interested in, given that YoGL is a period indicator derived from people's good person-years during the survey period, we construct a measure of physical health based on a chair stand test, in which respondents were asked to raise from a chair without using their arms, after confirming that they felt safe to do so. Respondents who did not feel safe to do the test or were unable to raise from the chair without using their arms were classified as not physically healthy. This health indicator was used for the results in Figure 2 of the main text as well as Figure S1 in the SI.

The results presented in Table 1 and Figures 3, 4, as well as S3 are again based on WVS, in combination with other data sources. The reason for this mix of data sources is that objective measures of physical limitations are not available in WVS, while SHARE and SAGE only provide objective measures for the population aged 50 and older in certain countries. MCSS, on the other hand, includes information on objective health for younger ages, yet only for a small sample of countries. Thus, in a first step we append SHARE and SAGE with MCSS. Second, we match all countries that are not available in SHARE, SAGE and/or MCSS with similar countries from those surveys. We match countries based on life expectancy at birth, as well as geographical proximity. Third, we regress tested health measures from SHARE and SAGE on age, gender, education and subjective health. Based on the estimated coefficients, we conduct out-of-sample predictions for the matched countries. This is possible since all predictor variables – age, gender, education and subjective health – are available in WVS. Due to the combination of different data sources, the results for the health dimension have to be taken with a grain of salt. We do believe, however, that the extrapolations allow us to demonstrate how YoGL can be calculated, once the required data is available.

3.3 Life Satisfaction

As described earlier, the subjective dimension in YoGL is the one that would be most difficult to derive from sources of information other than the direct assessment of the survey respondents themselves. By relying exclusively on surveys that already contain information on life satisfaction (WVS, SHARE), we avoid having to deal with the determinants of subjective wellbeing that can only be explained to a very small degree.

Figure S3 presents YoGL and its individual components at age 20 for women for 38 countries. The results are based on WVS data in combination with other data sources, as described above. A corresponding figure presenting the results for men can be found in the main text (Figure 3).

4. Sensitivity Analysis

Figure S4 provides the main results along with sensitivity analyses based on SHARE data, as discussed in Section "YoGL with complete data" in the main text.

Changing cut-off for physical health: For the main results (light blue bars), individuals who need to use their arms to stand up from a chair are considered to have physical limitations. Coding them as free from physical limitations leaves the results virtually unchanged (dark blue bars). The share of those who are simultaneously out-of-poverty, free from cognitive limitations, free from physical limitations and have positive life satisfaction increases from 85.9 percent to 86.2 percent given the more lenient threshold. Although YoGL increase slightly, changes are marginal and do not affect the country ranking.

Changing cut-off for cognitive health: The cut-off for cognitive limitations is originally set at two or more correct numeracy answers. If the threshold is instead set at three or more correct answers (yellow bar), the share of those who are simultaneously above all four threshold decreases from 85.9 percent to 76.6 percent and the country ranking varies slightly from the original estimations. Using memory instead of numeracy to assess cognitive ability (orange bar) also decreases the share of those who are simultaneously above all four cut-offs from 85.9 percent to 83.8 percent. Consequently, YoGL in all countries analysed for both women and men drop. Moreover, the country ranking differs slightly when memory is utilised instead of numeracy. The overall finding, however, holds for all operationalizations of cognitive ability: Southern as well as Central- and Eastern European countries have much lower YoGL than Western and Northern European countries.

Changing cut-off for life satisfaction: Different thresholds for positive life satisfaction also have a slight impact on the estimates, but do not alter the overall findings. A higher threshold for positive life satisfaction (6+, light gray bars) decreases the share of those reporting to have lived good years from 85.9 percent to 78.9 percent, yielding a reduction in YoGL. The overall finding that Southern as well as Central- and Eastern European countries have much lower YoGL than Western and Northern European countries, however, is again not affected. The only exception are German and French men, whose YoGL strongly depend on the cut-off set for positive life satisfaction. Changing the threshold from a rating larger than four to a rating larger than five decreases YoGL of German and French men stronger than YoGL of other groups. By contrast, a lower threshold for positive life satisfaction (4+, dark gray bars) increases the share of those who are simultaneously out-of-poverty, free from cognitive limitations, free from physical limitations and have positive life satisfaction increases from 85.9 to 87.5 percent and the country ranking remains almost identical.

In conclusion, the results and country rankings are generally quite robust to modifications of the cut-off points; however, these analyses show that the sensitivity is greater the closer the cut-off is to the middle of the underlying variable's distribution. This finding supports the choice to focus primarily on the tail ends of the distributions, i.e. those who are without doubt in very unfavorable conditions.

SI References

1. É. Laurent, *Measuring Tomorrow: Accounting for Well-Being, Resilience, and Sustainability in the Twenty-First Century* (Princeton University Press, 2017).

2. GDP at 70: What's Next? *The Globalist* (2014) (August 29, 2019).

3. D. Meadows, "Indicators and information systems for sustainable development" (Sustainability Institute, 1998).

4. P. Pradhan, L. Costa, D. Rybski, W. Lucht, J. P. Kropp, A systematic study of Sustainable Development Goal (SDG) interactions. *Earths Future* **5**, 1169–1179 (2017).

5. OECD Better Life Index (2013) (November 4, 2019).

6. J. Lorenz, C. Brauer, D. Lorenz, Rank-optimal weighting or "How to be best in the OECD Better Life Index?" *Soc. Indic. Res.* **134**, 75–92 (2017).

7. S. Ghislandi, W. C. Sanderson, S. Scherbov, A Simple Measure of Human Development: The Human Life Indicator. *Popul. Dev. Rev.* **45**, 219–233 (2019).

8. L. Osberg, A. Sharpe, An Index of Economic Well–Being for Selected OECD Countries. *Rev. Income Wealth* **48**, 291–316 (2002).

9. R. Jacobs, P. C. Smith, M. K. Goddard, *Measuring performance: an examination of composite performance indicators: a report for the Department of Health* (Centre of Health Economics, University of York, 2004).

10. K. Decancq, M. A. Lugo, Weights in Multidimensional Indices of Wellbeing: An Overview. *Econom. Rev.* **32**, 7–34 (2013).

11. M. C. Nussbaum, *Women and human development: The capabilities approach* (Cambridge University Press, 2001).

12. A. J. Robson, Group Selection: A Review Essay on *Does Altruism Exist?* by David Sloan Wilson. *J. Econ. Lit.* **55**, 1570–1582 (2017).

13. M. Fleurbaey, Beyond GDP: The Quest for a Measure of Social Welfare. *J. Econ. Lit.* **47**, 1029–1075 (2009).

14. A. Mascarenhas, P. Coelho, E. Subtil, T. B. Ramos, The role of common local indicators in regional sustainability assessment. *Ecol. Indic.* **10**, 646–656 (2010).

15. M. McGillivray, The human development index: Yet another redundant composite development indicator? *World Dev.* **19**, 1461–1468 (1991).

16. S. R. Chakravarty, *Analyzing Multidimensional Well-Being: A Quantitative Approach* (John Wiley & Sons, 2017).

17. J. E. Stiglitz, A. K. Sen, J.-P. Fitoussi, "Mismeasuring our lives: Why GDP doesn't add up" (The New Press, 2010).

18. E. Thorbecke, "Multidimensional Poverty: Conceptual and Measurement Issues" in *The Many Dimensions of Poverty*, N. Kakwani, J. Silber, Eds. (Palgrave Macmillan UK, 2013), pp. 3–19.

19. R. E. Johnson, C. C. Rosen, C.-H. Chang, To Aggregate or Not to Aggregate: Steps for Developing

and Validating Higher-Order Multidimensional Constructs. *J. Bus. Psychol.* **26**, 241–248 (2011).

20. R. Mukherjee, D. Sengupta, S. K. Sikdar, Parsimonious use of indicators for evaluating sustainability systems with multivariate statistical analyses. *Clean Technol. Environ. Policy* **15**, 699–706 (2013).
21. R. Veenhoven, Happy life-expectancy. *Soc. Indic. Res.* **39**, 1–58 (1996).
22. D. McLean, National and International indices of well-being: A critical analysis. *J. Indiana Acad. Soc. Sci.* **17**, 39–54 (2017).
23. S. Kuznets, *National Income, 1929-1932* (U.S. Government Printing Office, 1934).
24. J. Döpke, A. Knabe, C. Lang, P. Maschke, Multidimensional well-being and regional disparities in Europe. *JCMS J. Common Mark. Stud.* **55**, 1026–1044 (2017).
25. A. N. Menegaki, C. T. Tugcu, Energy consumption and Sustainable Economic Welfare in G7 countries: A comparison with the conventional nexus. *Renew. Sustain. Energy Rev.* **69**, 892–901 (2017).
26. A. C. Michalos, *Connecting the quality of life theory to health, well-being and education: The selected works of Alex C. Michalos* (Springer, 2017).
27. S. Abdallah, J. Michaelson, S. Shah, L. Stoll, N. Marks, "The Happy Planet Index: 2012 Report. A global index of sustainable well-being" (New Economics Foundation, 2012) (August 22, 2016).
28. S. Alkire, S. Jahan, "The New Global MPI 2018: Aligning with the Sustainable Development Goals" (OPHI Working Paper 121, University of Oxford. This paper is also cross …, 2018).
29. A. A. Lijadi, "Theoretical Foundations to outline Human Well-being: Metaanalytic Literature Review for defining Empowered Life Years" (International Institute for Applied Systems Analysis (IIASA), 2018).
30. P. Veneri, A. J. E. Edzes, Eds., REGION, The journal of ERSA - Special Issue: Well-being in cities and regions: measurement, analysis and policy practices. *Reg. J. ERSA* **4** (2017).
31. J. Boelhouwer, Bridging the gap: Overcoming data difficulties during 40 years of measuring well-being in The Netherlands. *Soc. Indic. Res.* **130**, 129–145 (2017).
32. A. E. Clark, A. J. Oswald, Satisfaction and comparison income. *J. Public Econ.* **61**, 359–381 (1996).
33. R. A. Easterlin, Life cycle happiness and its sources: Intersections of psychology, economics, and demography. *J. Econ. Psychol.* **27**, 463–482 (2006).
34. R. A. Easterlin, L. A. McVey, M. Switek, O. Sawangfa, J. S. Zweig, The happiness–income paradox revisited. *Proc. Natl. Acad. Sci.* **107**, 22463–22468 (2010).
35. E. Diener, Subjective well-being. *Psychol. Bull.* **95**, 542–575 (1984).
36. E. Diener, *et al.*, Findings all psychologists should know from the new science on subjective well-being. *Can. Psychol.* **58**, 87–104 (2017).
37. K. Czekierda, A. Banik, C. L. Park, A. Luszczynska, Meaning in life and physical health: systematic review and meta-analysis. *Health Psychol. Rev.* **11**, 387–418 (2017).
38. J. M. Smyth, M. J. Zawadzki, V. Juth, C. N. Sciamanna, Global life satisfaction predicts ambulatory affect, stress, and cortisol in daily life in working adults. *J. Behav. Med.* **40**, 320–331 (2017).

39. E. K. Defenderfer, T. M. Rybak, W. H. Davies, K. S. Berlin, Predicting parent health-related quality of life: evaluating conceptual models. *Qual. Life Res.* **26**, 1405–1415 (2017).

40. M. Răileanu Szeles, Comparative Examination of Self-Perceived Health and Other Measures of the Quality of Life Across the EU-27. *Soc. Indic. Res.* (2017) https:/doi.org/10.1007/s11205-017-1597-1 (December 11, 2017).

41. G. L. Wehby, B. W. Domingue, F. D. Wolinsky, Genetic Risks for Chronic Conditions: Implications for Long-term Wellbeing. *J. Gerontol. Ser. A* (2017) https:/doi.org/10.1093/gerona/glx154 (December 11, 2017).

42. R. Veenhoven, Happy life-expectancy: A comprehensive measure of quality-of-life in nations. *Soc. Indic. Res.* **39**, 1–58 (1996).

43. W. Lutz, A. Goujon, "Literate life expectancy: Charting the progress in human development" in *The End of World Population Growth in the 21st Century: New Challenges for Human Capital Formation and Sustainable Development*, W. Lutz, W. C. Sanderson, S. Scherbov, Eds. (Earthscan, 2004), pp. 159–186.

44. A. Chattopadhyay, K. C. Sinha, Spatial and Gender Scenario of Literate Life Expectancy at Birth in India. *Asia Pac. J. Public Health* **22**, 477–491 (2010).

45. M. C. Weinstein, G. Torrance, A. McGuire, QALYs: The Basics. *Value Health* **12**, S5–S9 (2009).

46. C. J. L. Murray, A. D. Lopez, Measuring the global burden of disease. *N. Engl. J. Med.* **369**, 448–457 (2013).

47. E. M. Crimmins, Y. Saito, D. Ingegneri, Trends in Disability-Free Life Expectancy in the United States, 1970-90. *Popul. Dev. Rev.* **23**, 555–572 (1997).

48. H. E. Klarman, J. O. Francis, G. D. Rosenthal, Cost Effectiveness Analysis Applied to the Treatment of Chronic Renal Disease. *Med. Care* **6**, 48–54 (1968).

49. R. Zeckhauser, D. Shepard, Where now for saving lives. *Law Contemp Probs* **40**, 5 (1976).

50. F. Sassi, Calculating QALYs, comparing QALY and DALY calculations. *Health Policy Plan.* **21**, 402–408 (2006).

51. W. D. Nordhaus, J. Tobin, Is Growth Obsolete? *Econ. Res. Retrosp. Prospect Vol. 5 Econ. Growth*, 1–80 (1972).

52. M. D. Morris, A physical quality of life index. *Spec. Issue Qual. Life* **3**, 225–240 (1978).

53. C. J. Murray, A. D. Lopez, Quantifying disability: data, methods and results. *Bull. World Health Organ.* **72**, 481 (1994).

54. Y. Yang, Long and happy living: Trends and patterns of happy life expectancy in the U.S., 1970-2000. *Soc. Sci. Res.* **37**, 1235–1252 (2008).

55. E. M. Crimmins, Y. Saito, D. Ingegneri, Trends in Disability-Free Life Expectancy in the United States, 1970-90. *Popul. Dev. Rev.* **23**, 555–572 (1997).

56. M. Fleurbaey, G. Gaulier, International Comparisons of Living Standards by Equivalent Incomes*. *Scand. J. Econ.* **111**, 597–624 (2009).

57. R. Boarini, F. Murtin, P. Schreyer, M. Fleurbaey, Multi-dimensional Living Standards: A Welfare Measure Based on Preferences. *OECD Stat. Work. Pap.* **2016** (2016).

58. C. I. Jones, P. J. Klenow, “Beyond GDP? Welfare across Countries and Time” (National Bureau of Economic Research, 2010) (February 16, 2015).

59. E. National Academies of Sciences and Medicine, *Leading Health Indicators 2030: Advancing Health, Equity, and Well-Being* (The National Academies Press, 2020) https:/doi.org/10.17226/25682.

60. New Zealand Government, Living Standards Framework Dashboard Update - December 2019 (2019) (September 21, 2020).

61. A. Börsch-Supan, Survey of Health, Ageing and Retirement in Europe (SHARE) Wave 5 (2019) https:/doi.org/10.6103/SHARE.w5.700 (May 10, 2019).

62. R. F. Inglehart, *et al.*, “World Values Survey: All Rounds - Country-Pooled Datafile 1981-2014” (JD Systems Institute, 2014).

63. P. Kowal, *et al.*, Data Resource Profile: The World Health Organization Study on global AGEing and adult health (SAGE). *Int. J. Epidemiol.* **41**, 1639–1649 (2012).

64. T. B. Üstün, *et al.*, WHO multi-country survey study on health and responsiveness. *Geneva World Health Organ.* (2001).

65. Eurostat, Life table (demo_mlifetable) (2020) (August 21, 2020).

66. United Nations, “World Population Prospects 2019” (Department of Economic and Social Affairs, Population Division, 2019).

67. D. P. Baker, J. Leon, E. G. Smith Greenaway, J. Collins, M. Movit, The education effect on population health: A reassessment. *Popul. Dev. Rev.* **37**, 307–332 (2011).

68. S. J. Olshansky, *et al.*, Differences in life expectancy due to race and educational differences are widening, and many may not catch up. *Health Aff. (Millwood)* **31**, 1803–1813 (2012).

69. Eurostat, “Life expectancy by age, sex and educational attainment level” (2013).

70. OECD, Divided we stand: Why inequality keeps rising (2011).

71. P. Hoeller, I. Joumard, I. Koske, *Income Inequality in OECD Countries: What are the Drivers and Policy Options?* (World Scientific, 2014).

72. World Bank, “World Development Indicators” (The World Bank, 2017).

73. Q.-L. Xue, J. D. Walston, L. P. Fried, B. A. Beamer, Prediction of Risk of Falling, Physical Disability, and Frailty by Rate of Decline in Grip Strength: The Women’s Health and Aging Study. *Arch. Intern. Med.* **171**, 1119–1121 (2011).

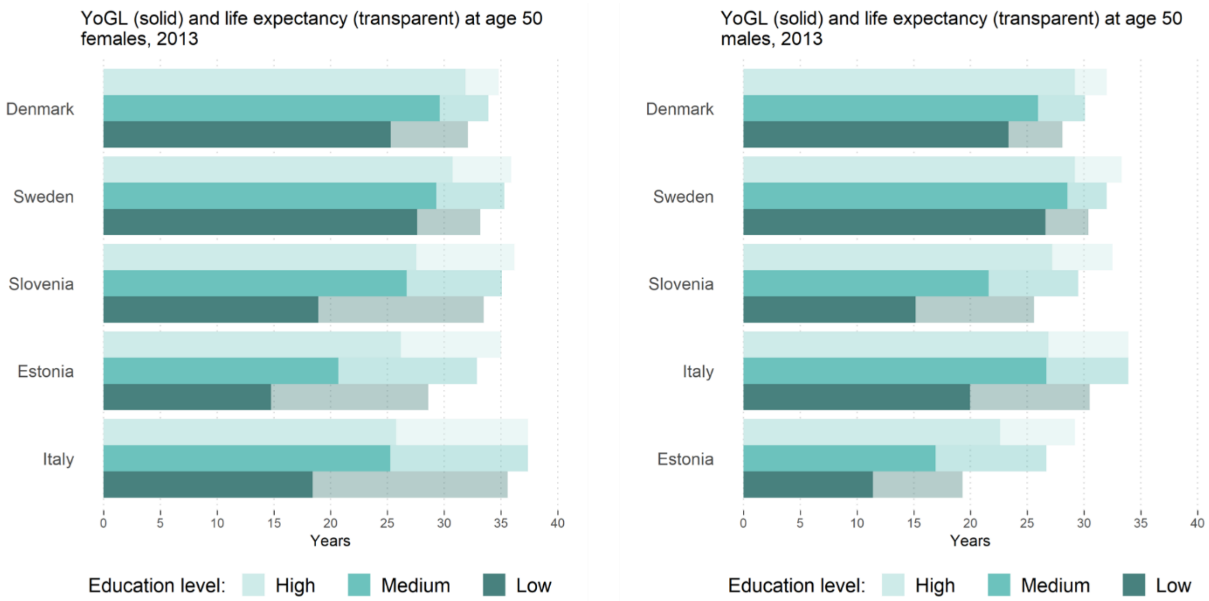

Figure S1. YoGL and life expectancy at age 50 by level of education. Selected European countries in 2013. Source: Own calculations based on SHARE and Eurostat.

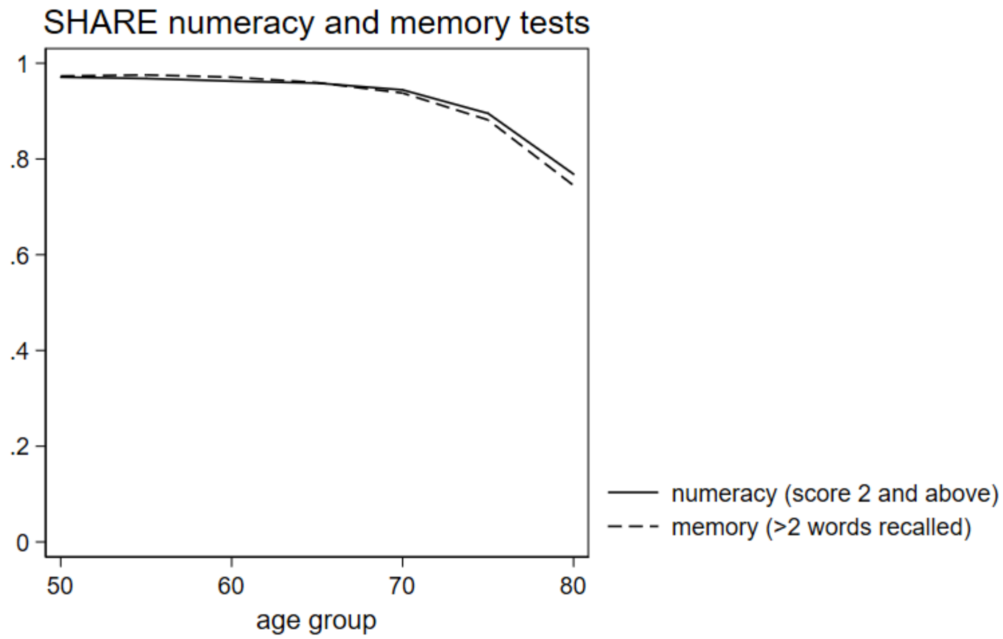

Figure S2. Comparison of age-specific measures of cognitive fitness. Source: SHARE.

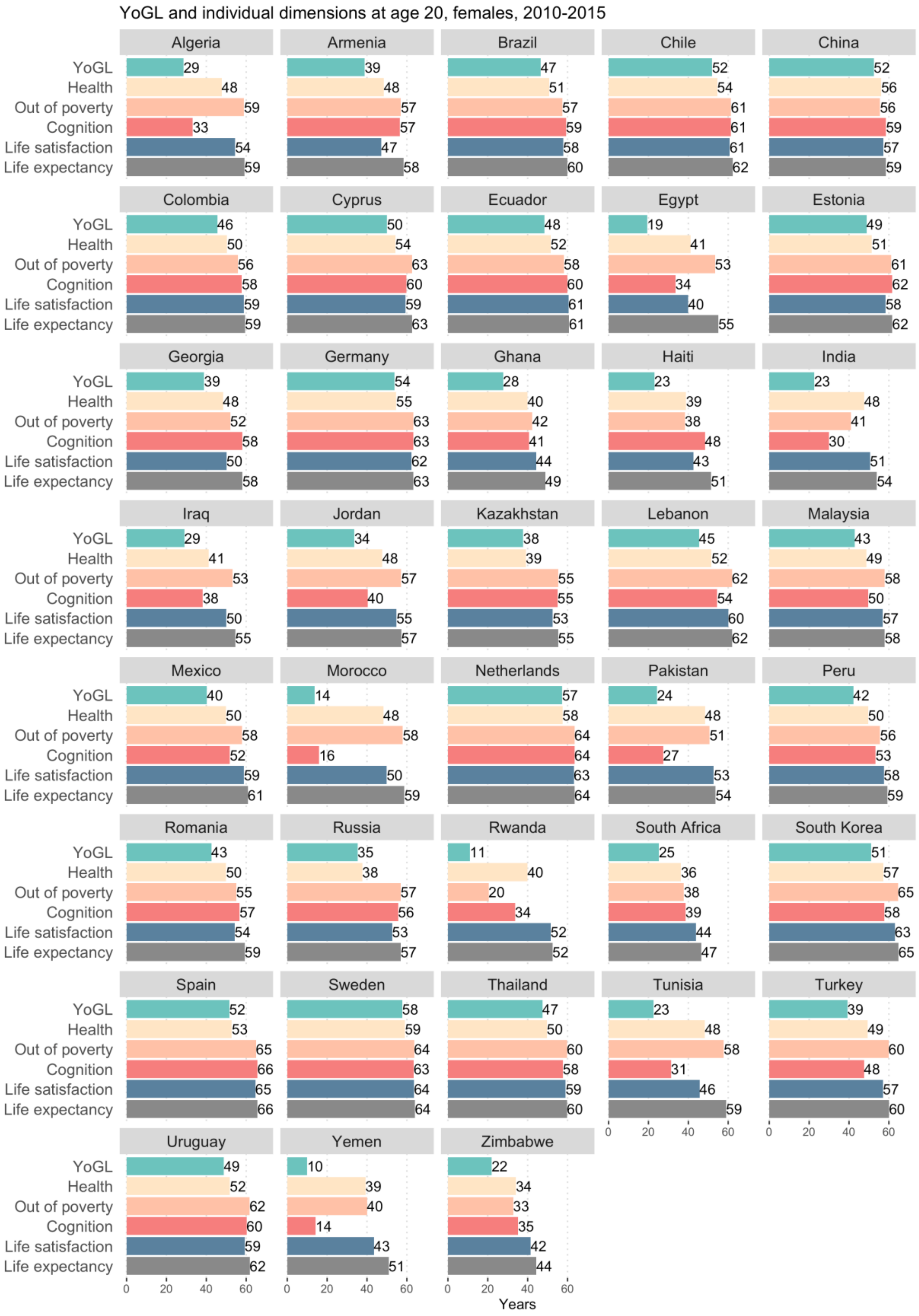

Figure S3. YoGL and its individual dimensions for 38 countries at age 20, females, 2010-2015.

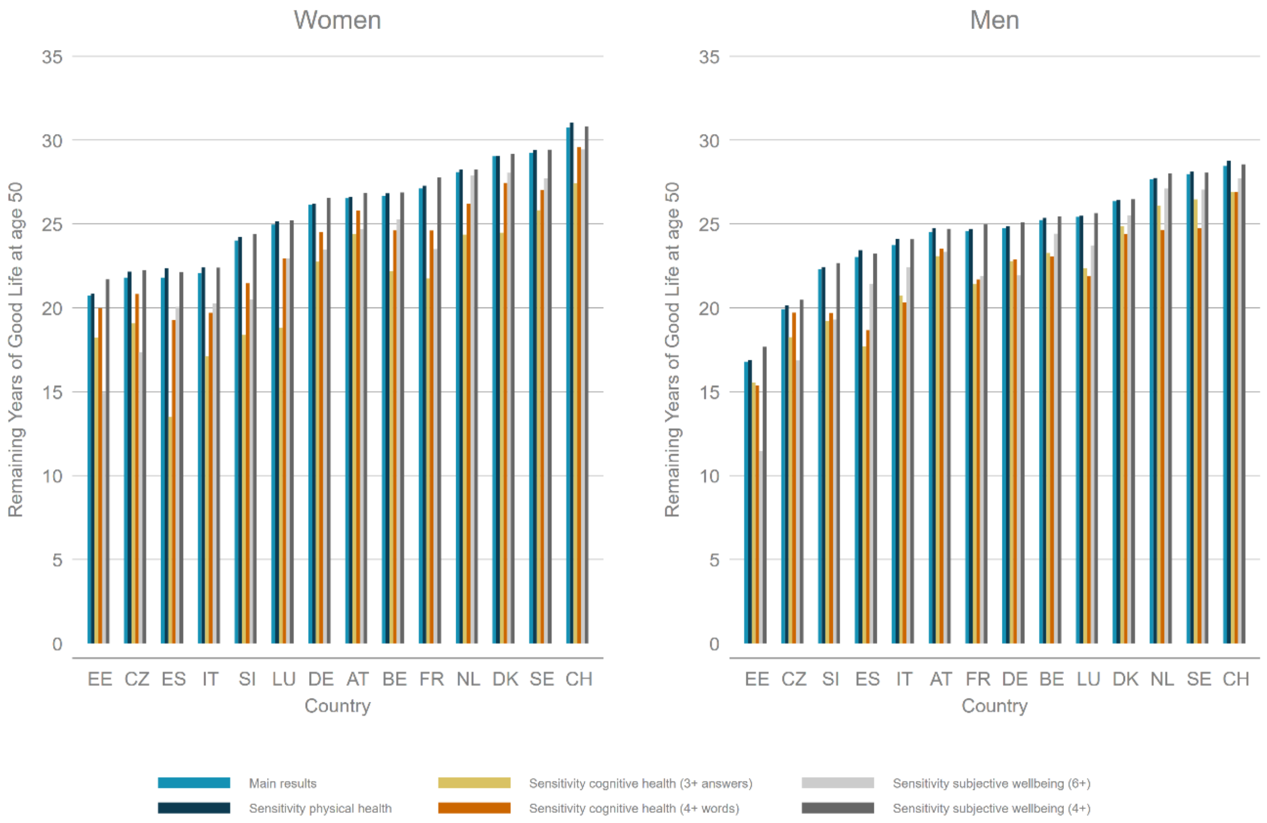

Figure S4. Sensitivity analyses for the remaining Years of Good Life at age 50 based on SHARE data (2013).
